# Proteome‐Wide Profiling of Olaparib Interactors Using a Biotinylated Photoaffinity Probe

**DOI:** 10.1002/cbic.202400882

**Published:** 2025-02-13

**Authors:** Femke L. A. M. van der Heijden, Suzanne A. Weijers, Onno Bleijerveld, Katarzyna W. Kliza, Michiel Vermeulen, Dmitri V. Filippov

**Affiliations:** ^1^ Leiden Institute of Chemistry Leiden University Einsteinweg 55, 2333 CC Leiden The Netherlands; ^2^ Division of Molecular Genetics The Netherlands Cancer Institute Plesmanlaan 121, 1066 CX Amsterdam The Netherlands; ^3^ Department of Molecular Biology, Faculty of Science, Radboud Institute for Molecular Life Sciences, Oncode Institute Radboud University Geert Grooteplein 28, 6525 GA Nijmegen The Netherlands; ^4^ Max Planck Institute of Molecular Physiology Otto-Hahn Strasse 11, 44227 Dortmund Germany

**Keywords:** Chemical Proteomics, Olaparib, PARP inhibitors, Photoaffinity labeling

## Abstract

Olaparib is a widely used PARP inhibitor for the treatment of BRCA‐mutated cancers. To comprehensively understand the drug‘s clinical impact, measuring its interactions with intended on‐ and off‐targets is crucial. In this study, olaparib‘s on‐ and off‐targets were profiled using photoaffinity labeling, a powerful, proteome‐wide method for studying the direct interactions between a drug and its protein targets. A novel photoaffinity probe was designed and used in a proteomic screening to discover novel targets of olaparib in the human proteome. The probe, incorporating a pre‐installed biotin group, bypasses the limitations of using a copper(I)‐catalyzed click reaction in cell lysates for reporter group conjugation and revealed a broad range of olaparib interactors, including previously unreported proteins, in a quantitative mass spectrometry‐based proteomic screening using HeLa whole cell lysate. This study contributes to our current understanding of the pharmacology of olaparib and provides a valuable tool for elucidating drug interactors within cell lysates, potentially guiding the development of more targeted therapeutics with fewer off‐targets.

## Graphical abstract

**Novel interactors found for olaparib**: Olaparib is an important anti‐cancer drug in BRCA‐mutated cancers. In this study, we identified novel interactors for olaparib using photoaffinity labeling. We designed a biotinylated photoaffinity probe and used it in a proteomic screening to discover a broad range of olaparib interactors, including previously unreported proteins, contributing to the current understanding of olaparib‘s pharmacology.

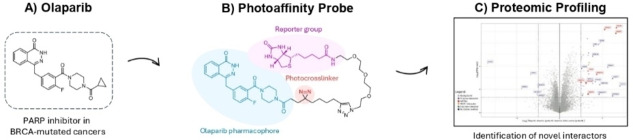

## 1 Introduction

Olaparib is a widely used drug for the treatment of BRCA‐mutated cancers, such as breast and ovarian cancer.[^1^, ^2^] Olaparib targets PARP1 and PARP2,[^3^, ^4^, ^5^] two members of the PARP enzyme family that consists of 17 ADP‐ribosyltransferases (ARTDs) that control the ADP‐ribosylation pathway.^[6]^ ADP‐ribosylation, mediated by PARP1 and PARP2, plays a crucial role in the DNA damage repair response.^[7]^ Because BRCA1/2‐mutated cancer cells are usually deficient in their homologous recombination pathways, they heavily rely on the ADP‐ribosylation signaling pathway for their DNA repair, making them uniquely vulnerable to PARP inhibitors, such as olaparib.^[2]^

To comprehensively understand the clinical impact of olaparib, it is crucial to determine the interaction of the drug with its intended on‐ and off‐targets.^[8]^ These interactors may include other members of the PARP family as well as additional proteins within the human proteome. A proteome‐wide method to identify the proteins that directly interact with a drug, both the intended targets and off‐targets, is photoaffinity labeling (PAL). PAL is a chemo‐proteomics technique that employs a photoaffinity probe composed of three key elements: a) a recognition element, which structurally mimics the drug and reversibly binds to relevant proteins, b) a photocrosslinker, which irreversibly binds to the protein upon exposure to light and c) a ligation handle, which can be attached to a fluorophore or biotin for visualization or affinity purification purposes, respectively.[^9^, ^10^, ^11^, ^12^]

**Figure 1:**
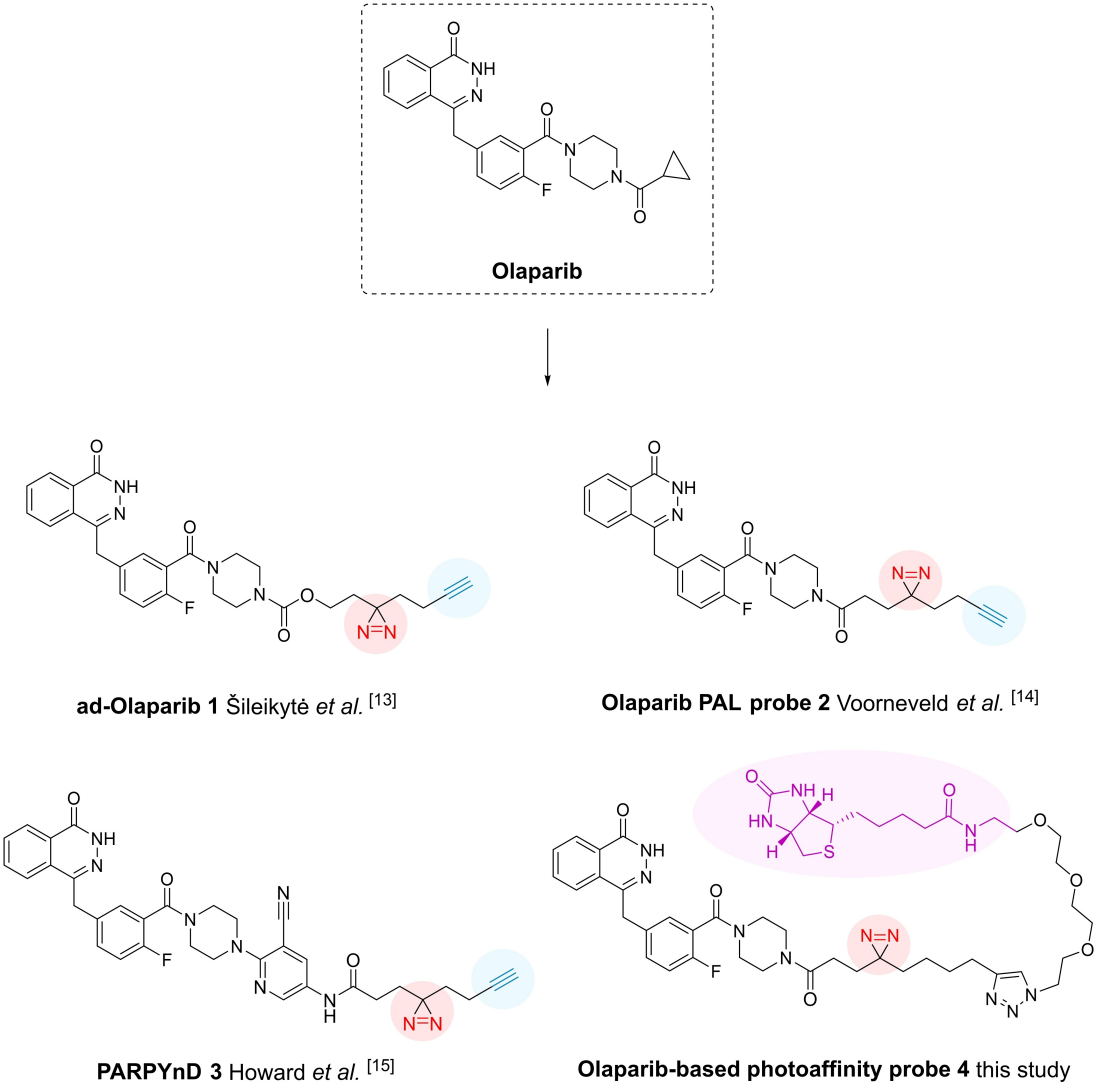
Overview of current photoaffinity probes derived from olaparib. Photocrosslinker (diazirine) is shown in red, ligation handle (alkyne) for conjugation to a reporter group is depicted in blue and the pre‐installed reporter group (biotin) is shown in purple. Olaparib‐based photoaffinity probe **4** is synthesized and applied in this study.

Recently, several studies have developed novel PAL probes derived from olaparib (probes **1**–**3**, Figure 1).[^13^, ^14^, ^15^] Notably, only one of these studies utilized the olaparib‐based photoaffinity probe to comprehensively identify and report the proteins targeted by olaparib at a proteome‐wide level. This study conducted proteomic analysis *in cellulo*, unveiling multiple previously unknown putative off‐targets for olaparib, including nicotinamide phosphoribosyltransferase (NAMPT), lanosterol synthase (LSS) and NADPH‐dependentΔ(24)‐sterol reductase (DHCR24).^[15]^ While this approach successfully identified numerous putative protein interactors for olaparib, the use of in‐cell target profiling suffers from limitations due to the need for an *in situ* copper(I)‐catalyzed click reaction to conjugate the reporter group to the probe‘s ligation handle.[^16^, ^17^] For example, degradation of peptides can occur due to the formation of reactive oxygen species (ROS) as a result of high concentrations of copper(I)[^18^, ^19^, ^20^] and low‐abundant targets may be missed due to the concentration‐dependent nature of the copper(I)‐catalyzed click reaction.^[18]^

**Figure 2:**
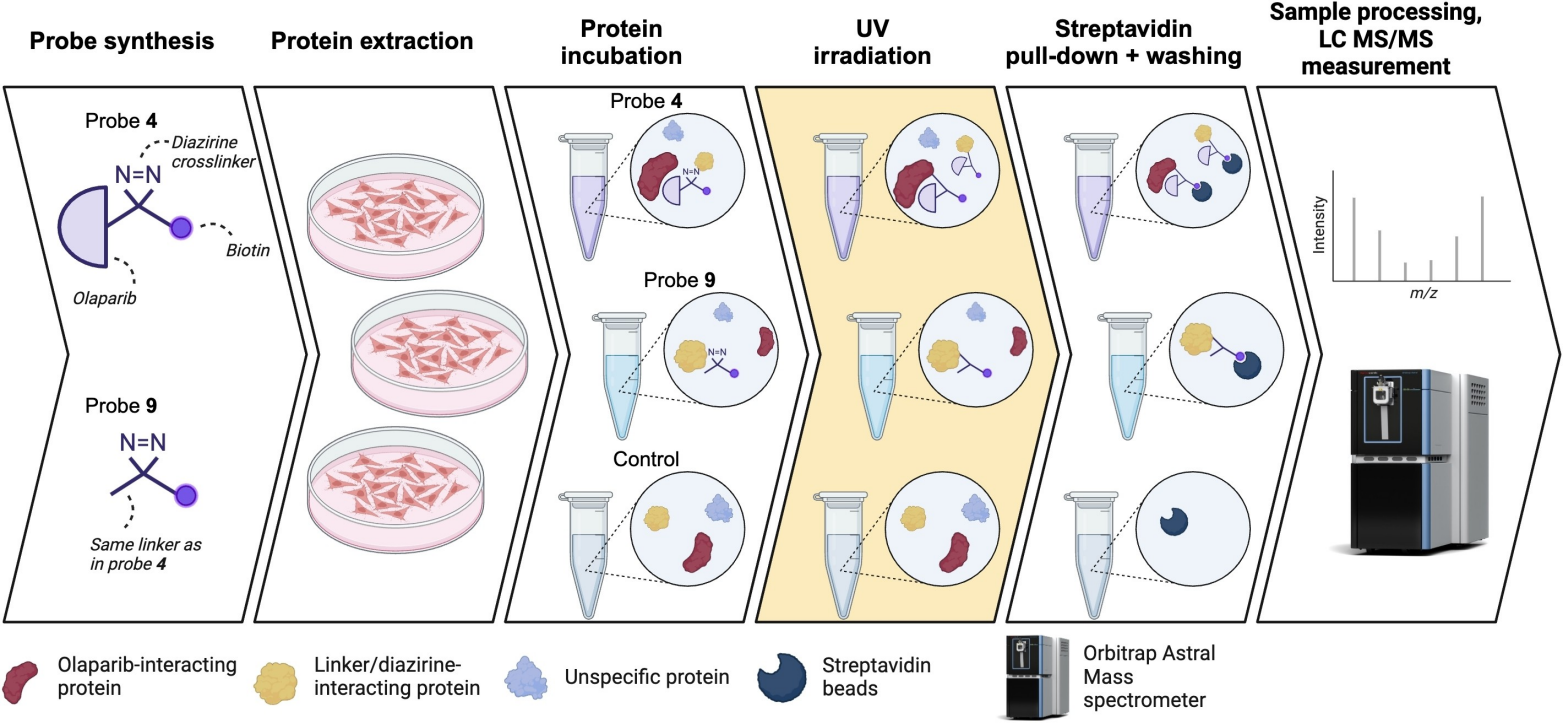
Workflow to identify direct on‐ and off‐targets of olaparib. Probe **4** is aimed to identify targets of olaparib, while probe **9** is devised to identify proteins that specifically bind to the linker, the diazirine and/or the biotin group. A direct comparison of proteins binding to each probe should result in determining the group of proteins that solely bind to olaparib. Empty streptavidin beads are used as an additional negative control. After incubation of each probe with the HeLa protein extract, samples are UV irradiated at 365 nm. This activates the diazirine group, directly crosslinking proteins in close proximity of the probe. Afterwards, the probe + protein complex is pulled down by streptavidin beads and samples are washed stringently to remove majority of non‐covalently bound proteins. Digested peptides are then measured and identified using an Orbitrap Astral mass spectrometer.

We hypothesized that employing a novel pre‐clicked photoaffinity olaparib probe in a proteomic analysis in cell lysates could serve as a valuable complementary approach to identify the protein targets of olaparib (probe **4**, Figure 1). Our probe design utilizes the distal nitrogen of the piperazine ring of olaparib to attach the diazirine‐linker, similar to previously published olaparib‐derived probes[^13^, ^14^, ^15^] and incorporates pre‐installed biotin as a reporter group, eliminating the need to use a copper(I)‐catalyzed click reaction in cell lysate for reporter group conjugation, which could diminish the sensitivity of the method. We applied this novel pre‐clicked olaparib probe **4** in a quantitative mass spectrometry‐based proteomics screening using HeLa whole cell lysate to discover novel targets of olaparib in the human proteome, thus contributing to our current understanding of the pharmacology of olaparib (Figure 2).

## 2 Results and Discussion

**Scheme 1:**
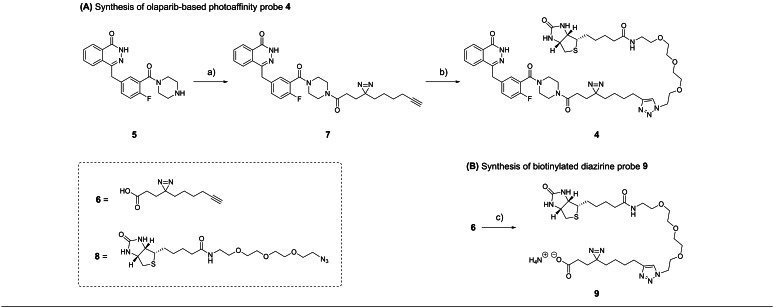
Synthetic scheme for the synthesis of **(A)** Olaparib‐based photoaffinity probe **4** and **(B)** Biotinylated diazirine probe **9**. Reaction conditions: a) **6**, DiPEA, HCTU, DMF, rt, overnight, 70 %. b) **8**, CuSO_4_, sodium ascorbate, THPTA, H_2_O/ACN, rt, 3 h, 7 %. c) **8**, CuSO_4_, sodium ascorbate, THPTA, H_2_O/ACN, rt, 2 h, 30 %.

First, photoaffinity‐based probe (AfBP) **4** derived from olaparib was synthesized via a two‐step procedure starting from commercially available olaparib precursor **5** (Scheme 1A). The first step was the HCTU‐mediated coupling of secondary amine **5** with known carboxylic acid **6**,^[21]^ producing intermediate **7** efficiently with a yield of 70 %. The obtained intermediate **7** containing both a terminal alkyne and a diazirine was subjected to a copper(I)‐catalyzed click reaction with commercially available biotin derivative **8** provided with an azide connected to biotin via a triethylene glycol linker. The reaction was followed by LC–MS analysis, and after reaction completion, AfBP **4** was successfully obtained with a yield of 7 % after purification by preparative RP‐HPLC followed by size‐exclusion chromatography.

In addition to olaparib‐based photoaffinity probe **4**, biotinylated diazirine probe **9** (Scheme 1B) was expeditiously obtained in a 30 % yield from alkyne **6** and azide **8** via a copper(I)‐catalyzed click reaction as described for **4**. Diazirine‐based probe **9** is an essential negative control in the proteomics analysis since it is known that the diazirine photoaffinity tag itself binds multiple proteins independently of the recognition element,^[22]^ which is, in this case, the olaparib pharmacophore. Thus, compound **9** is crucial to distinguish the binders of olaparib from those of the other components of the probe in the proteomics experiment.

To identify the on‐ and off‐targets of olaparib, probes **4** and **9** were first incubated with whole‐cell protein lysates from HeLa cells, a cervical cancer cell line naturally expressing multiple key components necessary for ADP‐ribosylation. This expression is essential for evaluating the functionality of probe **4** (Figure 2).^[23]^ After a 70‐minute incubation, during which the probes were protected from light, the samples were split into two groups: one which was exposed to UV irradiation and the other kept in the dark. Both samples were kept at 4 °C to prevent any unwanted enzymatic activity. Following this, a streptavidin pull‐down was performed, after which both samples were stringently washed. Bound proteins were then on‐bead digested with trypsin, after which tryptic peptides were analyzed using a data‐independent acquisition (DIA) method on an Orbitrap‐Astral mass spectrometer. Label‐free raw mass spectrometry data was analyzed using DIANN and further processed using Perseus and visualized in R.[^24^, ^25^]

**Figure 3:**
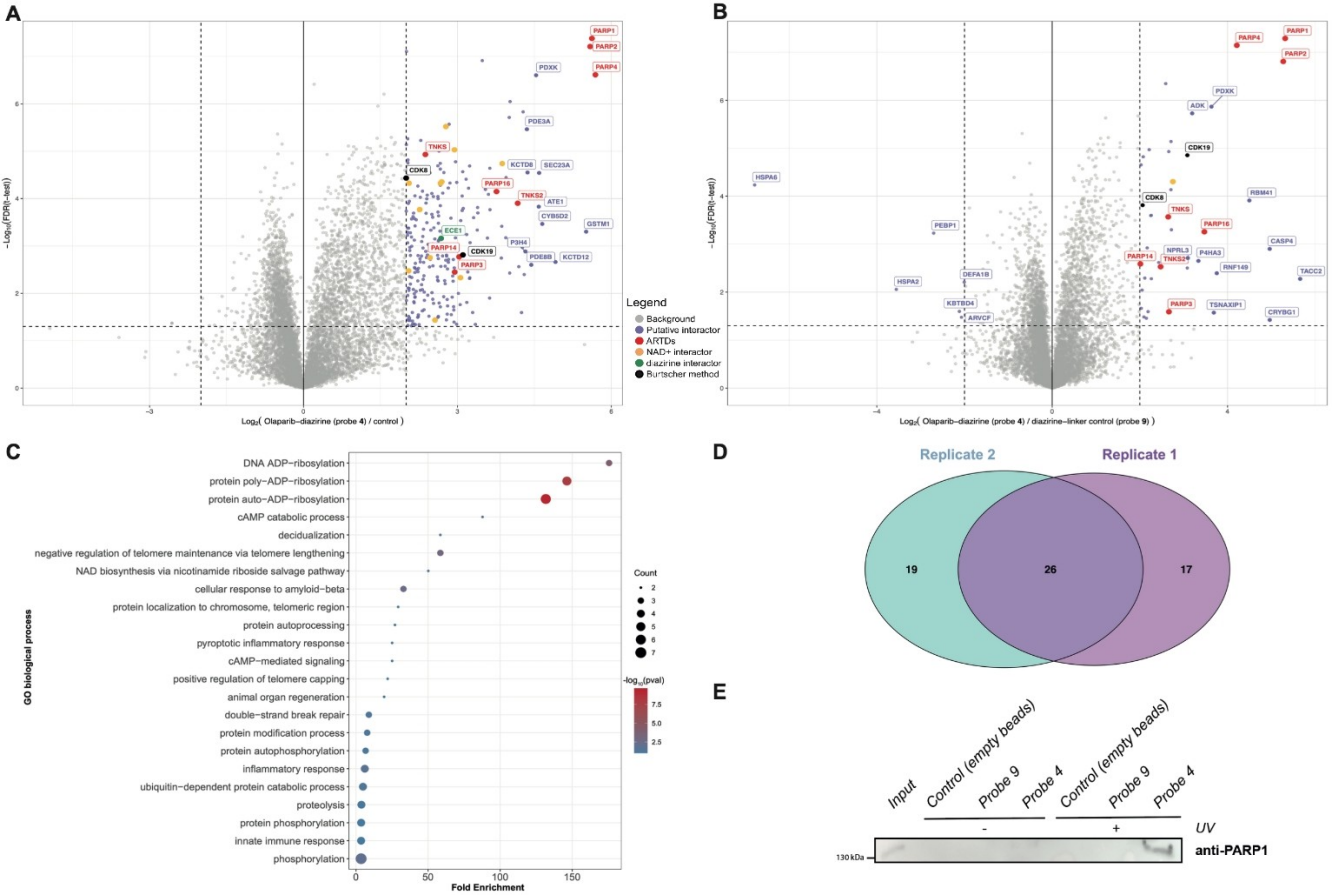
Affinity pull‐down with probe **4** and probe **9**. The experiment was performed with three technical replicates for each condition. **(A)** Volcano plot depicting preferential binding of proteins to probe **4** compared to the negative control (empty streptavidin beads). The statistical cutoffs in the t‐test are as follows: FDR <0.05 and fold change (FC)≥2. ARTDs, known NAD^+^ binding proteins, known diazirine interactors, and two kinases that were also identified by Burtcher *et al*.^[27]^ are indicated. Furthermore, the 10 putative interactors with the highest fold change are labeled. **(B)** Volcano plot depicting preferential binding of proteins to probe **4** over probe **9**. Statistical cutoffs are identical to (A). (A) and (B) share the same legend. **(C)** Dot plots depicting GO term enrichment analysis for proteins that bind significantly (p≤0.05, FC≥2) to probe **4** over probe **9**. GO terms are plotted against their fold enrichment. Color coding depicts the ‐Log_10_(p‐value) of statistically significant terms, dot size corresponds to protein count for each term. **(D)** Overlap between significantly enriched (same statistical cutoffs as in (A)‐(C)) proteins binding to probe **4** over probe **9** between two biological replicates in HeLa cells. Replicate 1 enriched for 43 proteins, replicate 2 for 45 proteins. **(E)** Western blot to validate interaction between the synthesized probes and PARP1.

The results demonstrated that probe **4** strongly enriches for PARP1 and PARP2, alongside six other ARTDs, when compared to empty streptavidin beads (Figure 3A). Given that many PARP inhibitors (PARPi), including olaparib, contain a benzamide pharmacophore designed to fit in the NAD^+^ binding pocket of PARP1, it was anticipated that other NAD^+^ binding proteins might also interact with this probe.^[26]^ Indeed, 10 of the proteins that significantly bound to probe **4** were classified as NAD^+^ binding proteins, excluding the ARTDs. Furthermore, the diazirine group in the probe is also known to specifically enrich for certain proteins, for which one was identified in this dataset.^[22]^

To discriminate bona fide olaparib binding proteins from diazirine or linker interactors, probe **9** was synthesized as a negative control bait (Figure 3B, Figure S4). About 80 % of the proteins identified in the experiment in which empty streptavidin beads were used as a negative control, were not identified when using probe **9** as a negative control, while ARTDs such as PARP1 and PARP2 were still significantly enriched by probe **4**. Gene Ontology enrichment analysis revealed that, in addition to ADP‐ribosylation, proteins interacting with probe **4** are involved in processes such as telomere maintenance, DNA repair, phosphorylation and various metabolic processes. This highlights that probe **4** selectively enriches proteins involved in key cellular processes, validating its utility for identifying olaparib interacting proteins (Figure 3C). This experiment was repeated in HeLa cells to serve as a biological replicate; both experiments consistently enriched for similar ARTDs (Figure 3D and Figure S1A–C).

We also validated the interaction between PARP1 and probe **4** using Western blotting (Figure 3E, Figure S2). To this end, the affinity purifications as described above were repeated, but this time, bound proteins were subjected to SDS‐page followed by immunoblotting using the respective antibody (Figure 2). The Western blot analysis confirmed a strong interaction between PARP1 and probe **4** following UV irradiation, further corroborating the mass spectrometry results on the interaction between PARP1 and our affinity‐based probe approach. A very small amount of PARP1 enrichment is visible for probe **4** even without UV irradiation, which could be attributed to the known strong interaction between PARP1 and olaparib or non‐covalent interactions not completely removed by stringent washing conditions.^[28]^ Nevertheless, PARP1 is still significantly more enriched to probe **4** after UV irradiation and stringent washing, confirming that PARP1 is covalently crosslinked to probe **4**.

To validate the specificity of the proteins enriched by probe **4**, a competitive binding assay was performed using unlabeled olaparib (AZD2281) as a competitor (Figure S5). HeLa whole cell protein extracts were pre‐incubated with a 1x (3 μM) or 100x (300 μM) molar excess of olaparib prior to incubation with probe **4**. Proteins preferentially enriched by probe **4** under no‐competition conditions were significantly reduced in abundance in the presence of increasing concentrations of olaparib. This concentration‐dependent competition demonstrates the specificity of these interactions for the olaparib pharmacophore, rather than non‐specific interactions with other components of probe **4**. Notably, ARTD's, including PARP1, PARP2, and PARP4, consistently show strong interaction with probe **4**, even at 1x molar excess, further confirming their specific binding to the olaparib‐derived probe.

The inhibitory activity of olaparib, probe **4**, and probe **9** on PARP1 was assessed in a PARP1 activity assay (Figure S6). The IC_50_ for olaparib was determined to be 0.014 μM, reflecting its high potency in inhibiting PARP1 activity. Probe **4** also inhibited PARP1, with an IC_50_ of 3.8 μM, indicating that while it is less potent that olaparib, it retains inhibitory functionality. In contrast, probe **9**, lacking the olaparib pharmacophore, did not exhibit significant PARP1 inhibition under the tested concentrations. Due to the addition of the biotin moiety, probe **4** is not cell‐permeable and cannot be tested in living cells, a limitation shared by similar photoaffinity probes.^[29]^ These results emphasize the strength of probe **4** in identifying interactions *in vitro*, making it a valuable addition to the growing arsenal of tools for investigating PARP inhibitors.

It is becoming increasingly clear that numerous off‐targets of several PARPi contribute to unwanted side effects and the development of resistance.[^30^, ^31^] Consequently, various methods have emerged to identify on‐ and off‐targets for PARPi, such as olaparib.[^3^, ^14^, ^15^, ^32^, ^33^] Interestingly, many of these previously reported olaparib off‐targets were not identified as significant interactors in our screening (Figure S3). Various reasons, such as the probe design or affinity purification conditions, may account for these discrepancies.

Recently, Burtcher *et al*. explored how different types of omics data can be integrated to provide a comprehensive view of the molecular mechanisms affected by PARP inhibition in ovarian cancer cells.^[27]^ The authors use the COSMOS framework, which connects data from Thermal Proteome Profiling (TPP), kinase activity (phosphoproteomics), and transcription factor activity (transcriptomics) to construct a network model that reveals the complex biological processes influenced by olaparib. The authors found a small overlap between the individual hits identified in each dataset, even within the context of related biological processes such as the DNA damage response. Despite limited overlap at the protein level, the integrated datasets through the COSMOS framework revealed critical biological insights that were not apparent when examining individual datasets, such as the discovery of novel signaling pathways and the connection between thermal stability and phosphorylation. Interestingly, two kinases identified in the COSMOS framework, CDK8 and its paralogue CDK19, both transcriptional regulators, were identified in our screening as direct olaparib interactors.^[34]^ These proteins remained enriched even after comparison with probe **9**, and were consistently found in both biological replicates (Figure 3A–B, Figure S1A–B). These results indicate that CDK8 and CDK19 may be biologically relevant olaparib off‐targets.

## 3 Conclusions

In conclusion, the synthesis of the novel pre‐clicked olaparib‐based probe **4** allowed for the efficient identification of both on‐ and off‐targets for olaparib. Probe **9**, which lacks the olaparib pharmacophore, serves as a critical control to distinguish specific olaparib interactors from proteins binding nonspecifically to the diazirine group or the linker. This approach, which bypasses the limitations of copper(I)‐catalyzed click reactions, revealed a broader range of olaparib interactors, including previously unreported proteins. This method thus provides a valuable tool for elucidating drug interactors within cell lysates, potentially guiding the development of more targeted therapeutics with fewer off‐targets.

## Supporting Information

The authors have cited additional references within the Supporting Information.[^35^, ^36^, ^37^]

## Conflict of Interests

The authors declare no conflict of interest.

4

## Supporting information

As a service to our authors and readers, this journal provides supporting information supplied by the authors. Such materials are peer reviewed and may be re‐organized for online delivery, but are not copy‐edited or typeset. Technical support issues arising from supporting information (other than missing files) should be addressed to the authors.

Supporting Information

## Data Availability

The data that support the findings of this study are available in the supplementary material of this article. The raw mass spectrometry proteomics data have been deposited to the ProteomeXchange Consortium via the PRIDE partner repository with the dataset identifier PXD: PXD060372.
