# Supplementary material for: Proteome‐Wide Profiling of Olaparib Interactors Using a Biotinylated Photoaffinity Probe
Source: Chembiochem. 2025 Feb 13;26(6):e202400882. doi: 10.1002/cbic.202400882 (PMC11907390; doi:10.1002/cbic.202400882)
Supplement: Supplementary file 1 — Supporting Information [file CBIC-26-e202400882-s001.pdf]

ChemBioChem

Supporting Information

Proteome-Wide Profiling of Olaparib Interactors Using a Biotinylated Photoaffinity Probe

Femke L. A. M. van der Heijden, Suzanne A. Weijers, Onno Bleijerveld, Katarzyna W. Kliza, Michiel Vermeulen,* and Dmitri V. Filippov*

Supporting Information

Proteome-Wide Profiling of Olaparib Interactors Using a Biotinylated Photoaffinity Probe

Femke L. A. M. van der Heijden+,[a] Suzanne A. Weijers+,[b,c], Onno Bleijerveld,[b] Katarzyna W. Kliza,[c,d] Michiel Vermeulen*,[b,c] Dmitri V. Filippov*[a]

Table of Contents

1. Additional Figures S2
2. Experimental Section S7
3. Characterization Data S12

1. Additional Figures

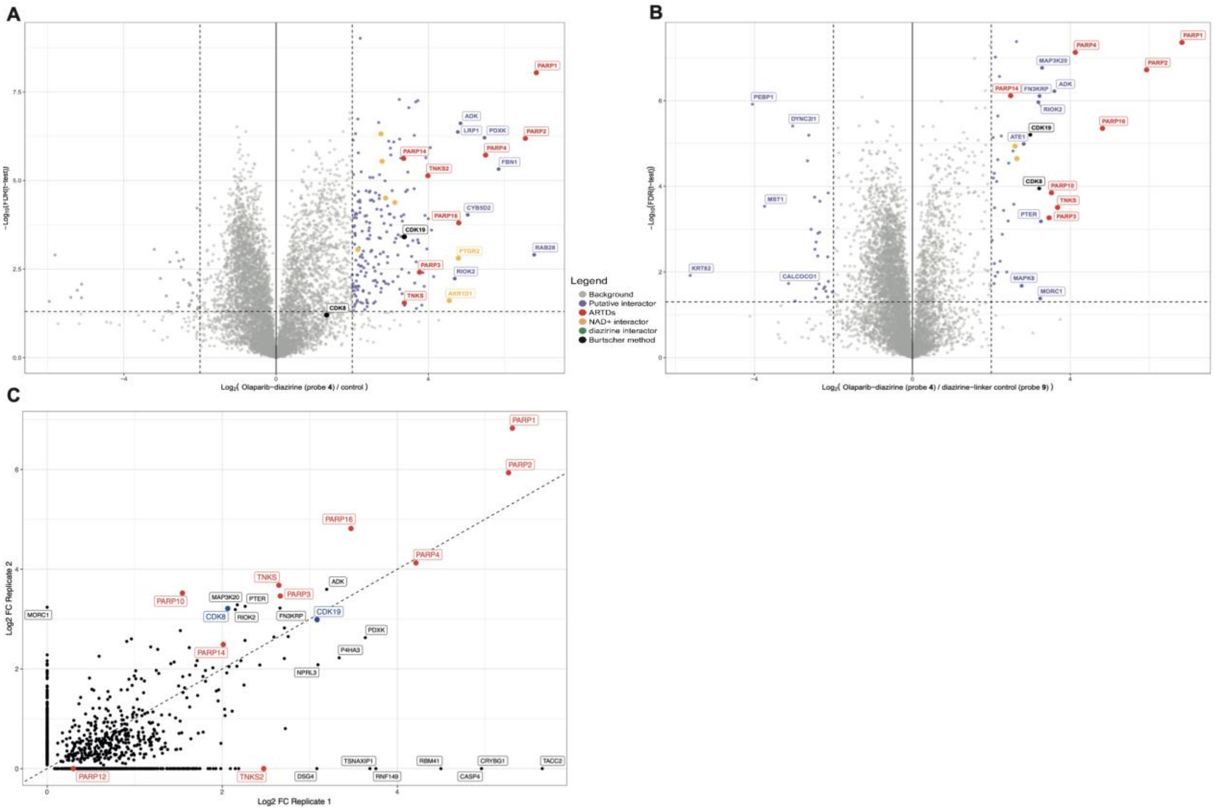

Figure S1. Affinity pull-down with probe **4** and probe **9**, replicate 2. **(A)** Volcano plot depicting preferential binding of proteins to probe **4** compared to the negative control (empty streptavidin beads). The statistical cutoffs in the t-test are as follows: FDR <0.05 and fold change (FC) ≥ 2. ARTDs, known NAD$^+$ binding proteins, known diazirine interactors, and two kinases that were also identified by Burtcher *et al.* are indicated. Furthermore, the 10 putative interactors with the highest fold change are labeled. **(B)** Volcano plot depicting preferential binding of proteins to probe **4** over probe **9.** Statistical cutoffs are identical to (A). (A) and (B) share the same legend. **(C)** Correlation plot depicting the statistically enriched proteins (FDR < 0.05, FC ≥ 0) between replicate 1 and replicate 2. ARTDs are indicated in red, CDK's identified by Burtscher *et al.* are indicated in blue. Proteins with a fold change higher than 3 are indicated in black.

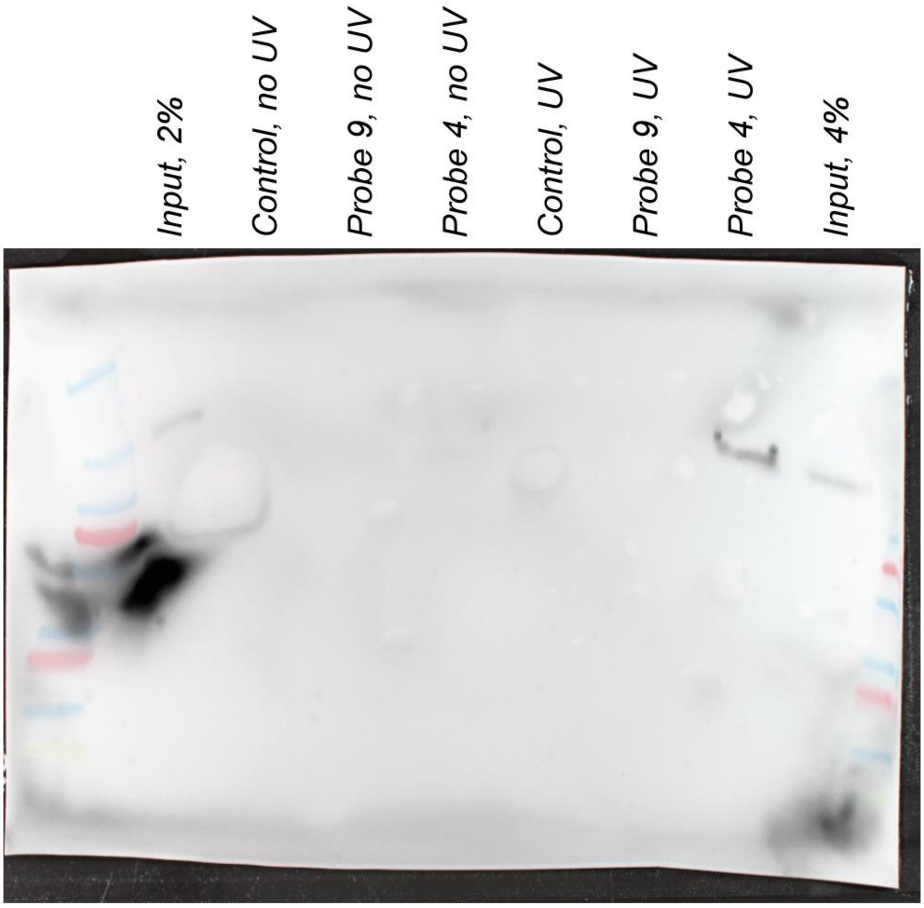

Figure S2. Uncropped western blot membrane as shown in figure 3E. Blotted for PARP1 enrichment. Control means incubation with empty streptavidin beads, instead of streptavidin beads bound to probe **4** or **9**.

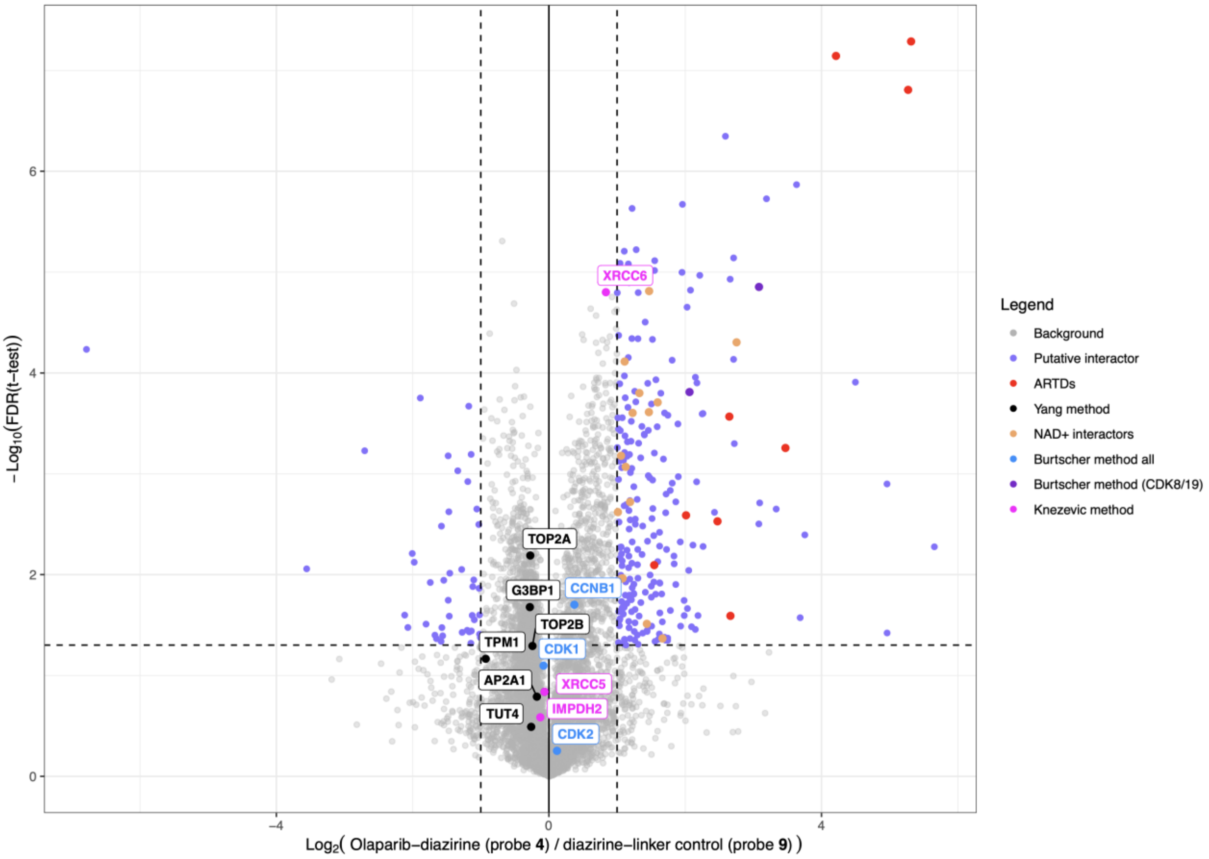

Figure S3. Examples of proteins identified in several methods to study on- and off-targets of olaparib, but not found to significantly bind to probe **4** (FDR < 0.05, FC ≥ 1). Same figure as Fig. 3B, but with the labels removed for the putative interactors, NAD$^+$ binding proteins, diazirine interactors, and the kinases identified by Burtscher *et al.* and here.

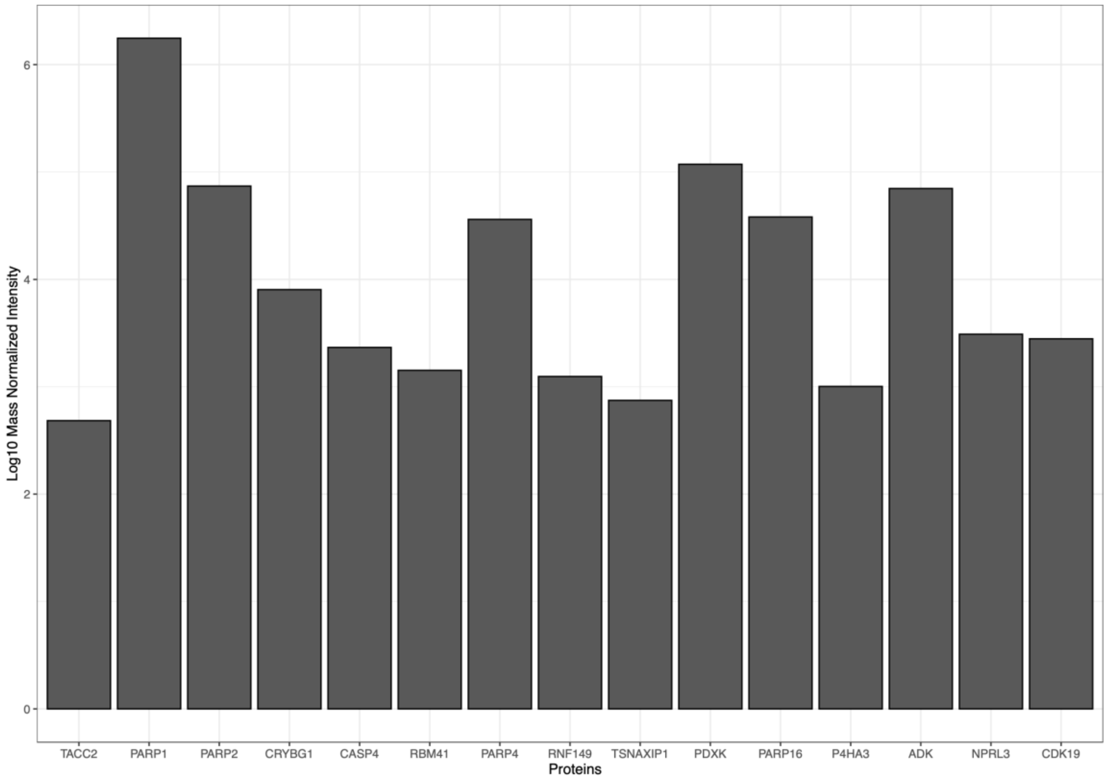

Figure S4. Abundances of the top 15 proteins to interact with probe **4**, as shown in figure 3B. Proteins are ranked based on their Log2 fold change, in descending order. Mass Normalized Intensity is determined by dividing the sum raw intensity for each protein group by their number of theoretically observable peptides. The latter is determined by performing an in silico trypsin digestion.

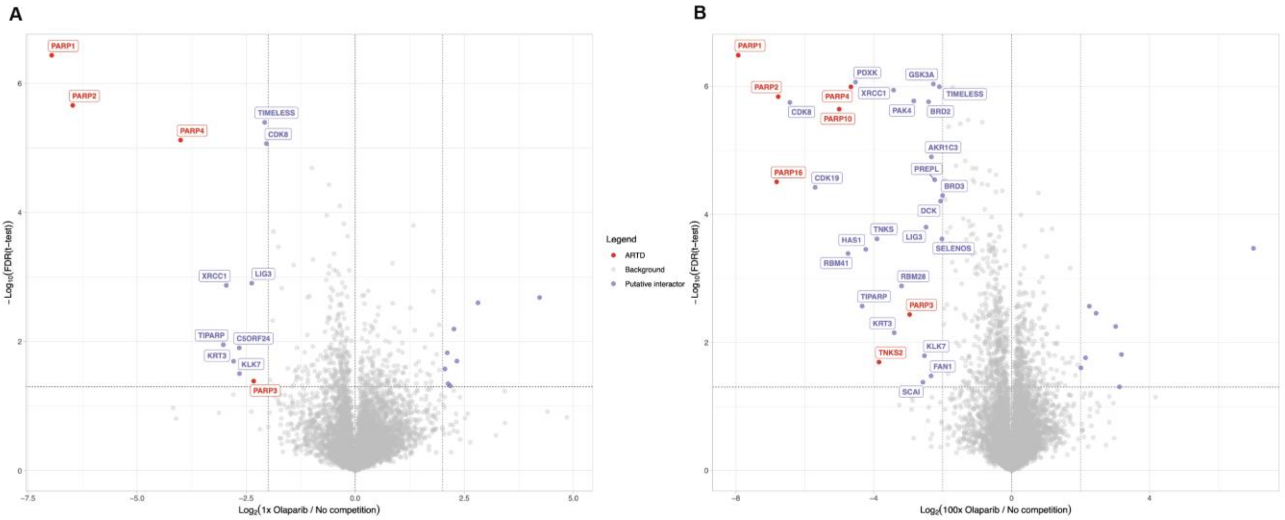

Figure S5. Competitive binding assay with olaparib (AZD2281). HeLa whole cell protein extract was pre-incubated with olaparib at 3µM (1:1 competition, 1x) or 300µM (100:1 competition, 100x) for 30 minutes. Subsequently, the extract was incubated with 3µM probe 4 for 70 minutes, followed by the protocol outlined for Figure 3A-B and Figure S1. **(A)** Volcano plot showing preferential binding of proteins to probe **4** under no-competition conditions versus competition with 1x olaparib. **(B)** Volcano plot showing preferential binding of proteins to probe **4** under no-competition conditions versus competition with 100x olaparib. In both panels, statistical cutoffs for the t-test were set at FDR <0.05 and fold change (FC) ≥ 2. ARTDs are highlighted in red, and the legend applies to both figures.

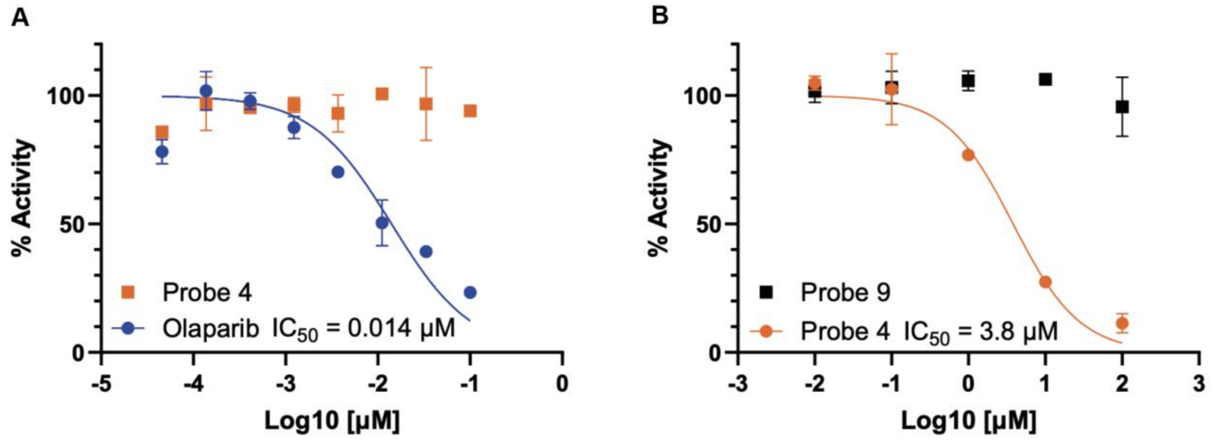

Figure S6. PARP1 inhibition assay. **(A)** Concentration-response curves of PARP1 activity when incubated with increasing concentrations of **(A)** olaparib or probe **4** or **(B)** probe **4** or probe **9**. All samples were tested in duplicates. IC_{50} values were derived using a nonlinear regression model in GraphPad Prism 10.

2. Experimental Section

General

All chemicals were of reagent grade and were used without further purification. All reactions were performed under an N_2 atmosphere unless stated otherwise. Reactions were monitored by analytical thin layer chromatography (TLC) using Merck aluminum sheets pre-coated with silica gel 60Å with detection by UV-absorption (254 nm) and by spraying with a solution of $KMnO_4$ (20 g/L) and K_2CO_3 (10 g/L) in water or ninhydrin (0.75 g/L) in EtOH followed by charring. Additional analysis with TLC-MS was used when needed. Column chromatography was performed manually using Macherey-Nagel silica gel 60Å (40-63 µm) in the indicated solvents. For HW-40 gel filtration purifications an ÄKTA explorer system equipped with a Superdex-30-HR column (16 mm x 60 cm, flow 1 mL/min) was used. For reversed-phase preparative HPLC purifications a Gilson HPLC system equipped with a C18 semi-preparative column (Gemini C18, 250x10 mm, 5 µm particle size, Phenomenex, flow 5 mL/min) was used.

NMR spectra were recorded on a Bruker AV-400, AV-500 or AV-850 spectrometer. Chemical shifts are given in ppm (*∂*) relative to the chloroform, acetonitrile-d_3 or deuterium oxide residual solvent peak or tetramethylsilane (TMS) as an internal standard. Coupling constants (*J*) are given in Hz. All given ^{13}C-NMR spectra are proton decoupled. 2D NMR experiments (HSQC, COSY) were carried out to assign protons and carbons of the synthesized structures.

High resolution mass spectra (HRMS) of the new compounds were recorded with a Q-Exactive HF Orbitrap (Thermo Scientific) equipped with an electrospray ion source (ESI) and injection of 2 µl of a 1 µM solution via an Ultimate 3000 nano UPLC (Dionex) system with an external calibration (Thermo Scientific), source voltage of 3,5 kV, capillary temperature of 275 °C, no sheath gas, resolution R = 240.000 at m/z=400 (mass range m/z=160-2000 or until a maximum of 6000) and ACN:H_2O (1:1 v/v) supplemented with 0.1% formic acid as eluent. LC-MS analysis was performed on an LCQ Advantage Max (Thermo Finnigan) ion-trap spectrometer (ESI+) coupled to a Surveyor HPLC system (Thermo Finnigan) equipped with a C18 column (Gemini, 4.6 mm x 50 mm, 3 µM particle size, Phenomenex) equipped with buffers A: H_2O, B: acetonitrile (ACN), C: 1% TFA or on an Agilent technologies 1260 infinity LC-MS with a 6120 Quadrupole MS system equipped with buffers A: H_2O, B: acetonitrile (ACN) and C: 100 mM NH_4OAc.

4-(4-Fluoro-3-(4-(3-(3-(hex-5-yn-1-yl)-3*H*-diazirin-3-yl)propanoyl)piperazine-1-carbonyl)benzyl)phthalazin-1(2*H*)-one (7)

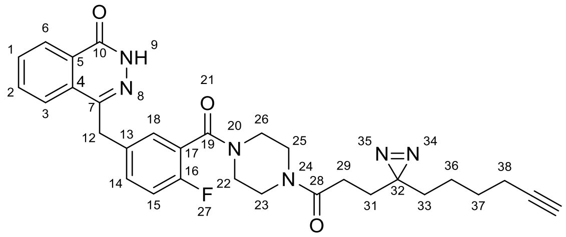

Amine **5** (47 mg, 0.13 mmol) was dissolved in DMF (1.3 mL). To this solution, carboxylic acid **6**[21] (27 mg, 0.14 mmol, 1.1 eq), DiPEA (49 µL, 0.28 mmol, 2.2 eq) and HCTU (58 mg, 0.14 mmol, 1.1 eq) were added and the reaction mixture was stirred at room temperature overnight. Then, the reaction was quenched with H_2O and the aqueous phase was extracted with EtOAc (3x). The combined organic layers were dried over $MgSO_4$, filtered and concentrated under reduced pressure. Purification by silica gel column chromatography (1% → 5% MeOH in DCM) afforded amide **7** as a white foam (48 mg, 0.09 mmol, 70%). **^{1}H NMR (400 MHz, $CDCl_3$)** δ 10.85 – 10.72 (m, 1H, H9), 8.48 (d, *J* = 5.7 Hz, 1H, H6), 7.80 – 7.70 (m, 3H, H1, H2, H3), 7.38 – 7.31 (m, 2H, H14, H18), 7.08 – 7.02 (m, 1H, H15), 4.30 (s, 2H, H12), 3.83 – 3.25 (m, 8H, H22, H23, H25, H26), 2.19 – 2.13 (m, 2H, H36), 2.07 (t, *J* = 7.6 Hz, 1H, CH alkyne), 2.01 – 1.93 (m, 2H, H37), 1.83 (t, *J* = 7.5 Hz, 2H, H38), 1.49 – 1.41 (m, 4H, H29, H33), 1.26 – 1.24 (m, 2H, H31). **^{13}C NMR (101 MHz, $CDCl_3$)** δ 170.1, 169.9 (C28 *rotamers*), 165.4, 165.2 (C19 *rotamers*), 160.6 (C10), 158.4, 155.9 (C16 *rotamers*), 145.7 (C7), 134.6 (C13), 133.9 (C1/2/3), 132.0, 131.9 (C14 *rotamers*), 131.8 (C1/2/3), 129.7 (C4), 129.5, 129.3 (C18 *rotamers*), 128.4 (C5), 127.3 (C6), 125.2 (C1/2/3), 123.8, 123.6 (C17 *rotamers*), 116.5, 116.4, 116.3, 116.2 (C15 *rotamers*), 84.1 (Cq. alkyne), 68.8 (CH alkyne), 47.1, 46.9, 45.7, 45.1, 42.2, 42.1, 42.0, 41.5 (C22, C23, C25, C26), 37.8 (C12), 32.7 (C29), 28.0 (C38), 28.0 (C33), 27.1 (C37), 23.1 (C31), 18.3 (C36). **HRMS** (ESI) $[M+H]^+$ calculated for $C_{30}H_{32}FN_6O_3$ 543.25144; found 543.25126.

Olaparib-based photoaffinity probe (4)

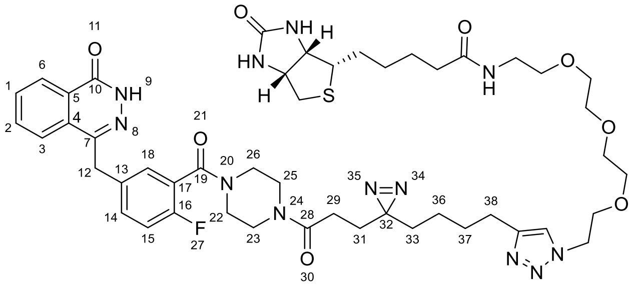

Alkyne **7** (4.74 mg, 8.74 µmol) was dissolved in H_2O (514 µL). Next, azide-PEG$_3$-biotin **8** (0.056 M in ACN, 312 µL, 17.48 µmol, 2 eq) was added and the mixture was diluted with additional H_2O (500 µL). Then, pre-mixed click cocktail (1:1:1 v/v/v, $CuSO_4$ (26 mg/mL in H_2O): sodium ascorbate (120 mg/mL in H_2O): THPTA (44 mg/mL in H_2O), 314 µL) was added and the reaction mixture was shaken at room temperature for 3 hours until LC-MS analysis indicated complete conversion. Next, the reaction mixture was quenched with EDTA (0.5 M in H_2O, 80 µL) and purification by reversed-phase preparative HPLC (A: 50 mM NH_4HCO_3 in milliQ, B: acetonitrile, gradient: 0-10% B) followed by HW-40 gel filtration (0.15 M NH_4OAc in 10% ACN/MilliQ) afforded probe **4** as a white solid (0.60 mg, 0.61 µmol, 7%) after repeated lyophilization. **^{1}H NMR (850 MHz, CD$_3$CN)** δ 11.22 – 11.11 (m, 1H, H9), 8.32 (dd, *J* = 11.6, 7.7 Hz, 1H, H6), 7.83 – 7.81 (m, 2H, H2, H3), 7.80 – 7.77 (m, 1H, H1), 7.58 – 7.57 (m, 1H, CH triazole), 7.43 – 7.39 (m, 1H, H14), 7.25 – 7.23 (m, 1H, H18), 7.13 (td, *J* = 9.0, 4.5 Hz, 1H, H15), 6.61 (s, 1H, NH amide biotin), 5.47 – 5.41 (m, 1H, NH carbamide biotin), 5.20 – 5.07 (m, 1H, NH carbamide biotin), 4.44 (dt, *J* = 15.5, 5.1 Hz, 2H, CH_2 PEG), 4.41 (t, *J* = 6.4 Hz, 1H, CH biotin), 4.31 (s, 2H, H12), 4.23 (ddd, *J* = 7.8, 4.4, 1.9 Hz, 1H, CH biotin), 3.81 (dt, *J* = 10.4, 5.1 Hz, 2H, CH_2 PEG), 3.65 (t, *J* = 5.4 Hz, 1H), 3.61 – 3.57 (m, 1H), 3.55 – 3.50 (m, 9H, CH_2 PEG), 3.47 (t, *J* = 5.3 Hz, 1H), 3.44 (t, *J* = 5.6, 1.5 Hz, 2H, CH_2 PEG), 3.38 (t, *J* = 5.3 Hz, 1H), 3.28 – 3.24 (m, 3H, CH_2 PEG), 3.18 – 3.12 (m, 3H, CH-S), 2.87 (AB, *J* = 12.7, 5.0 Hz, 1H, CH_2-S), 2.64 – 2.60 (m, 3H, CH_2-S), 2.14 – 2.09 (m, 4H), 1.67 – 1.53 (m, 8H), 1.44 – 1.41 (m, 2H), 1.38 – 1.35 (m, 2H), 1.15 – 1.11 (m, 2H). **^{13}C NMR (214 MHz, CD$_3$CN)** δ 173.8 (CO amide biotin), 171.0, 170.9 (C28 *rotamers*), 165.6, 165.5 (C19 *rotamers*), 163.9 (CO carbamide biotin), 160.9 (C10), 158.3, 157.0 (C16 *rotamers*), 146.3 (C7), 135.9, 135.8 (C13 *rotamers*), 134.4 (C2/3), 132.6, 132.5 (C14 *rotamers)*, 132.5 (C1), 130.5 (Cq. triazole), 129.9, 129.8, 129.7 (C18 *rotamers)*, 129.4 (C4), 129.4 (C5), 127.3, 127.3 (C6 *rotamers*), 126.5, 126.4 (C2/3 *rotamers*), 125.0 (C17), 122.9 (CH triazole), 117.0, 116.9, 116.9, 116.8 (C15 *rotamers)*, 71.1, 71.0, 70.9, 70.3, 70.1 (CH_2 PEG), 62.4, 60.8 (CH biotin), 56.3 (CH-S), 50.7 (CH_2 PEG), 47.6, 47.5, 46.0, 45.6, 42.5, 42.4, 42.3, 41.8, 41.2 (CH_2-S), 39.8 (CH_2 PEG), 38.0, 37.9 (C12 *rotamers*), 36.3, 33.0, 32.9, 29.7, 29.7, 29.5, 29.1, 29.0, 29.0, 29.0, 27.7, 27.7, 26.4, 25.9, 24.1, 24.0. **LC-MS** (10→50% ACN [0.1% TFA]): Rt = 6.9 min, m/z: 987.4 **HRMS** (ESI) $[M+H]^+$ calculated for $C_{48}H_{64}FN_{12}O_8S$ 987.46693; found 987.46673.

Biotinylated diazirine probe (9)

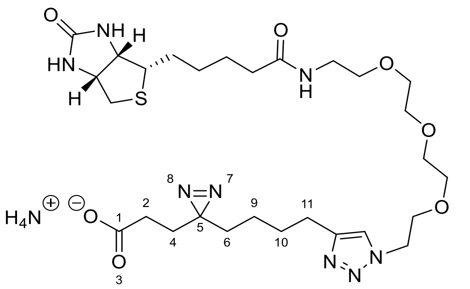

Alkyne **6**[21] (0.90 mg, 4.62 µmol) was dissolved in H_2O (272 µL). To the resulting solution, azide-PEG$_3$-biotin **8** (0.056 M in ACN, 165 µL, 9.24 µmol, 2 eq) was added, and the mixture was diluted with additional H_2O (693 µL). Then, pre-mixed click cocktail (1:1:1 v/v/v, $CuSO_4$ (26 mg/mL in H_2O): sodium ascorbate (120 mg/mL in H_2O): THPTA (44 mg/mL in H_2O), 166 µL) was added and the reaction mixture was shaken at room temperature for 2 hours until LC-MS analysis indicated complete conversion. Next, the reaction mixture was quenched with EDTA (0.5 M in H_2O, 42 µL) and purification by reversed-phase preparative HPLC (A: 50 mM NH_4OAc in milliQ, B: acetonitrile, gradient: 30-50% B) afforded probe **9** as a white solid (0.90 mg, 1.37 µmol, 30%) after repeated lyophilization. **^{1}H NMR (500 MHz, D$_2$O)** δ 7.83 (s, 1H, CH triazole), 4.62 – 4.57 (m, 3H, CH biotin, CH_2 PEG), 4.41 (dd, *J* = 8.0, 4.5 Hz, 1H, CH biotin), 3.97 (t, *J* = 5.0 Hz, 2H, CH_2 PEG), 3.69 – 3.56 (m, 10H, CH_2 PEG), 3.38 (t, *J* = 5.3 Hz, 2H, CH_2 PEG), 3.31 (dt, *J* = 10.0, 5.4 Hz, 1H, CH-S), 2.98 (AB, *J* = 13.0, 5.1 Hz, 1H, CH_2-S), 2.77 (d, *J* = 13.1 Hz, 1H, CH_2-S), 2.68 (t, *J* = 7.4 Hz, 2H, H11), 2.26 (t, *J* = 7.3 Hz, 2H, CH_2 biotin),

2.15 (t, J = 7.4 Hz, 2H, H2), 1.71 (t, J = 7.4 Hz, 2H, H4), 1.65 – 1.53 (m, 6H, CH_2 biotin, H10), 1.46 (t, J = 8.1 Hz, 2H, H6), 1.43 – 1.36 (m, 2H, CH_2 biotin), 1.11 (p, J = 7.8 Hz, 2H, H9). **LC-MS** (0→50% ACN [10 mM NH_4OAc]): Rt = 7.5 min, m/z: 611.4. **HRMS** (ESI) $[M+H]^+$ calculated for $C_{28}H_{47}N_8O_7S$ 639.32829; found 639.32812.

Cell culture

Human cervix adenocarcinoma HeLa cells were cultured at 37°C with 5% CO_2 in Dulbecco's Modified Eagle's Medium (DMEM, Thermo), supplemented with 1% Penicillin/Streptomycin (15140-122, Thermo) and 10% Fetal bovine serum (FBS, Serana). HeLa cells were routinely checked and tested negative for *Mycoplasma* contamination.

Preparation of whole cell extract

Cells were harvested at 80-90% confluency by trypsinization (trypsin, Thermo), and cell pellet (centrifugation at 400g for 5 minutes at 4°C) was washed three times with ice-cold 1x PBS. All subsequent steps were also performed on ice or at 4°C. Cells were lysed in 2.5x the pellet volume of ice-cold lysis buffer (50mM Tris-HCl pH 8.0, 150mM NaCl, 1.5mM $MgCl_2$, 0.5% NP40, 1µl benzonase (Sigma-Aldrich), 1x Complete Protease Inhibitor Cocktail, 1x PhosStop (Sigma-Aldrich), and 1mM DTT). Resuspended cells were passed five times through a syringe and incubated rotating for 30 minutes. Lysed cells were centrifuged for 30 minutes at 10000 rpm, and supernatant was transferred and kept aside in a new tube. The remaining pellet of unlysed cells was resuspended in 0.5x the original pellet volume with the lysis buffer and passed 10 times through a syringe. 1µl of benzonase was added and was incubated for 30 minutes in rotation. This was followed by 30 minutes centrifugation at 10000rpm, supernatant was then added to the tube with the previously collected supernatant. The lysate was filtered using a 0.22µm filter and syringe. This was centrifuged again for 10 minutes at 10000rpm. The supernatant was collected followed by addition of EDTA to a final concentration of 2mM and glycerol to 10% v/v. Concentration of the protein extract was measured with a BCA assay, and aliquots were snap frozen and stored at -80°C.

UV irradiation and subsequent streptavidin pull-down

To prepare protein extract for incubation with each probe, 1.5mg whole cell protein extract was incubated with binding buffer (50mM Tris-HCl pH 8.0, 150mM NaCl, 2mM EDTA, 0.1% NP40, Complete protease inhibitor (#04693132001, Roche), PhosSTOP™ (4906845001, Roche), and 0.5mM DTT) and additional Complete Protease Inhibitor and PhosStop to a 1x dilution for each sample. Each sample was prepared in technical triplicates, for label-free quantification purposes. Lysate mix was left on ice for 15 minutes, which was followed by incubation with 3µM of probe **4**, probe **9**, or an equal amount of buffer that each probe was dissolved in (10mM Tris-HCl pH 8.0, and 2mM EDTA) as control. During this 70-minute incubation all falcons were rotated and kept in the dark to prevent any residual light exciting the probe. Each sample was then divided in half: the +UV and -UV condition. +UV samples were UV irradiated for 20 minutes at 365nm for 20 minutes using an RMR-600 Photochemical Reactor (Rayonet), while the -UV samples were kept in the dark, all at 4°C. Afterwards, samples were equilibrated on ice for 5 minutes, which was followed by incubation with streptavidin beads (Cytiva, Streptavidin Sepharose High performance #17511301) for 30 minutes in rotation. Samples were then stringently washed, all at room temperature: first two times with a harsh wash buffer (8M of urea, 50mM of Tris-HCl pH 8.0, 1% NP40, complete protease inhibitor, and PhosStop), each time 5 minutes rotating. This was followed by two washes with the binding buffer, each wash rotating at room temperature for 5 minutes as well. Finally, to remove all residual salts and detergents, samples were washed four times at room temperature with 1x PBS. Bound proteins in each sample were then subjected to on-bead digestion with trypsin as described in the "On-bead digestion" section, or used for analysis with Immunoblotting as described in the "Immunoblotting" section.

Competitive binding assay

1mg of whole cell protein extract was pre-incubated with either a 1x (3µM) or 100x (300µM) molar excess of olaparib (AZD1288, Selleckchem, #S1060) for 30 minutes at 4°C in binding buffer. Subsequently, pre-incubated extracts were incubated with 3µM probe **4** for 70 minutes, followed by the protocol as described in the section “UV irradiation and subsequent streptavidin pull-down”. All samples were exposed to UV irradiation and each sample was again prepared in technical triplicates.

On-bead digestion

On-bead trypsin digestion was performed as previously described.[35] In short, remaining supernatant was removed from the beads using 30G syringes, followed by elution with a buffer (2M urea, 50mM Tris pH 8.5, 10mM DTT) and incubated at room temperature in a ThermoMixer set to 1200rpm for 20 minutes. The alkylating agent iodacetamide (Sigma-aldrich) was then added to a final concentration of 55mM and incubated in the dark for 10 minutes, 1200rpm. This was followed by the addition of 250ng of trypsin to each sample, and incubated for 2 hours, again at 1200rpm. Samples were centrifuged for 2 minutes at 2000rpm and the supernatant was collected. For a second round of elution again the elution buffer was added to the beads and incubated for 10 minutes in a thermoshaker at 1200rpm. The eluate was again collected after centrifugation and added to the previous eluate. This was followed by adding an additional 200ng of trypsin to each sample for digestion, and overnight incubation at room temperature. The following day samples were acidified to a pH below 2 with 10% v/v trifluoroacetic acid and peptide desalting using StageTips.

Immunoblotting

Proteins were separated by SDS-PAGE, using a 4-15% TGX gradient gel (Bio-rad), and transferred to a 0.22µm nitrocellulose membrane (iBlot 3.0 transfer stacks, Invitrogen) by dry transfer using the iBlot 3.0 system (Invitrogen) on the broad range setting. Membranes were blocked with 5% milk in non-fat dried milk in PBS-T and probed overnight with the primary antibody (rabbit polyclonal anti-PARP antibody, Cell Signaling #5942) at 4°C. The following day, membranes were washed thrice with PBS-T and incubated with the appropriate HRP-conjugated secondary antibody for 1 hour at room temperature. Membranes were then washed six times with PBS-T and then imaged using a Fusion Fx (Vilber).

Label-free quantification by LC-MS/MS

Peptides were reconstituted in 0.1% formic acid and analyzed by nanoLC-MS/MS on an Orbitrap Astral mass spectrometer (Thermo Scientific) connected to a Vanquish Neo nano-LCsystem (Thermo Scientific). The Vanquish Neo was operated in the trap-and-elute mode and peptides were loaded onto a Pepmap 100 C18 5µm trap column (300µm x 5mm, ThermoScientific), before separation on the analytical column (AUR325075C18TS, 1.7µm/75µm x 250mm, Thermo Scientific) mounted into an Easyspray ion source (Thermo Scientific) with 1600V spray voltage applied. The column was heated at 50^0C and the flow rate was set to 0.5µL/min at the start of the method to minimize delay time. Solvent A was 0.1% formic acid/water and solvent B was 0.1% formic acid/80% acetonitrile. Peptides were eluted at a flow rate of 0.4 µL/min in a 36-min effective gradient, containing a non-linear increase from 1% to 45% solvent B and a 0.4-min ramp to 99% solvent B at 0.5 µL/min flow rate at the end. The column was washed for 3.9 min. at 0.5 µL/min at 99%B and finally equilibrated using the "fast equilibration" script in combined control mode with a 1500 bar pressure limit. The Orbitrap Astral was run in data-independent acquisition (DIA) mode, with full MS scans being collected in the Orbitrap analyzer with 240,000 resolution at *m/z* 200 over a 380-980 *m/z* range. Default charge state was 2+, the normalized AGC target was set to 500% (equivalent to 5e6 charges) and the maximum injection time was set to 5 ms. For DIA MS2, a normalized HCD collision energy of 25% was applied to a 380-980 m/z precursor range using non-overlapping isolation windows of 2Th, with window placement optimization turned on. Scans were acquired in the Astral analyzer over a 100-1000 *m/z* range, with the normalized AGC target set to 500% (equivalent to 5e4 charges) with a maximum injection time of 3 ms.

Mass spectrometry data analysis

All raw mass spectrometry spectra were processed using DIA-NN software (version 1.8.0 or later) according to developers guidelines.[24] In Perseus, MaxLFQ intensity values from DIA-NN were $\log_2$ transformed, followed by filtering to detect proteins present in all replicates of at least one experimental triplicate (version 1.6.15.0).[25] Missing values in the dataset were imputed following a normal distribution (shift = 1.8 and width = 0.3) with the assumption that these proteins were just below the detection limit. Data was visualized in volcano plots by applying a two-sample t-test analysis (FDR ≤ 0.05, FC ≥ 1). Final data visualization of volcano plots and the Venn diagram were done in R.

The Gene Ontology enrichment analysis was performed using DAVID (v2024q2) on the significantly enriched proteins to probe **4** in replicate 1.[36,37] As a background list all the proteins detected in the experiment were used. Statistically enriched terms were identified by using the default settings in DAVID. Data visualization as a dot plot was done in R.

PARP1 inhibition assay

The inhibitory effect of probe 4, probe 9 and olaparib on PARP1 activity was tested with a chemiluminescence-based PARP1 assay kit (BPS bioscience, #80580) in two independent experiments (each compound in duplicate), following the instructions of the manufacturer. In short, wells of a 96-wells plate were coated with a histone mixture at 4 °C overnight. After the addition of activated DNA, biotinylated NAD^+, and the PARP1 enzyme, the ADP-ribosylation reaction was initiated for 60 minutes at room temperature. Biotinylated PAR was quantified with the use of a streptavidin-HRP and the addition of a colorimetric HRP substrate. Absorbance of the wells were recorded at 450nm with a microplate reader (Tecan), and the IC_{50} values were calculated with Graphpad Prism 10 software.

3. Characterization Data

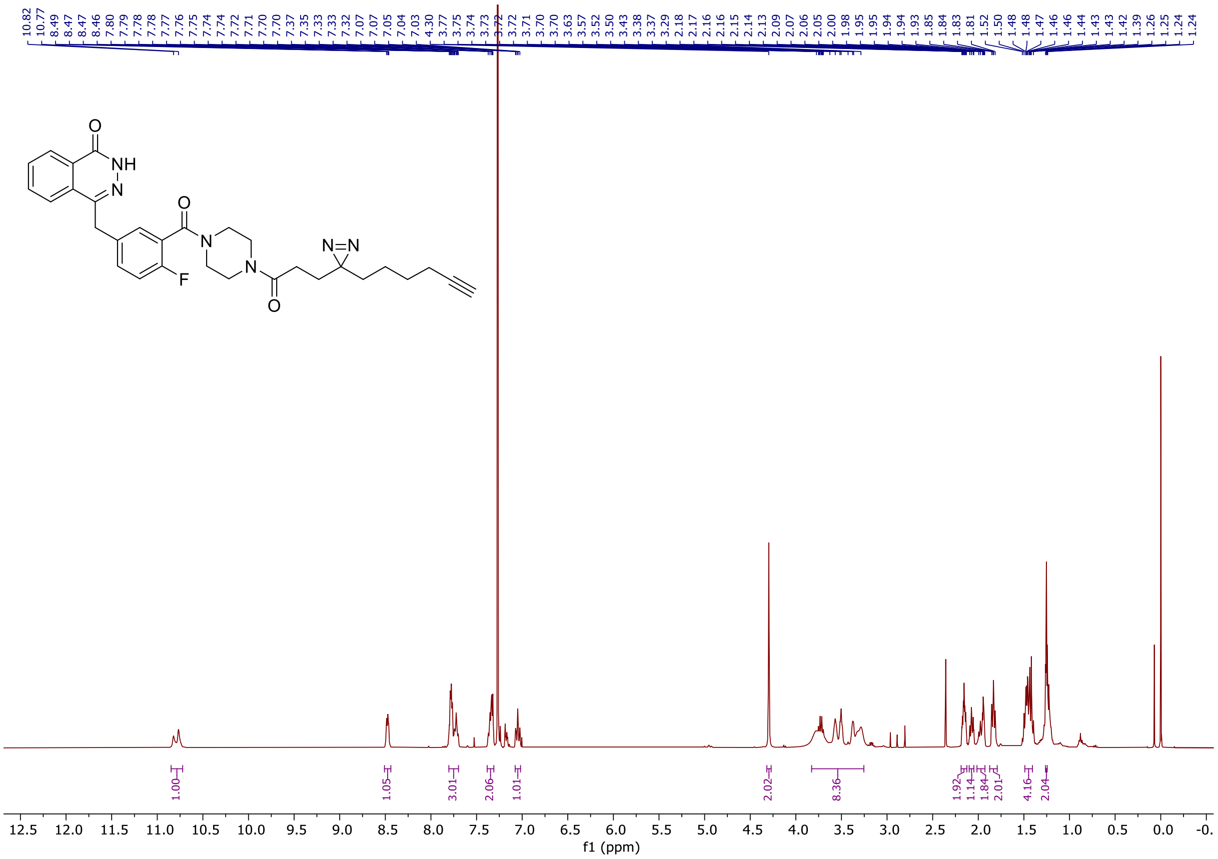

Figure S7. ^{1}H NMR Spectrum of Compound **7**

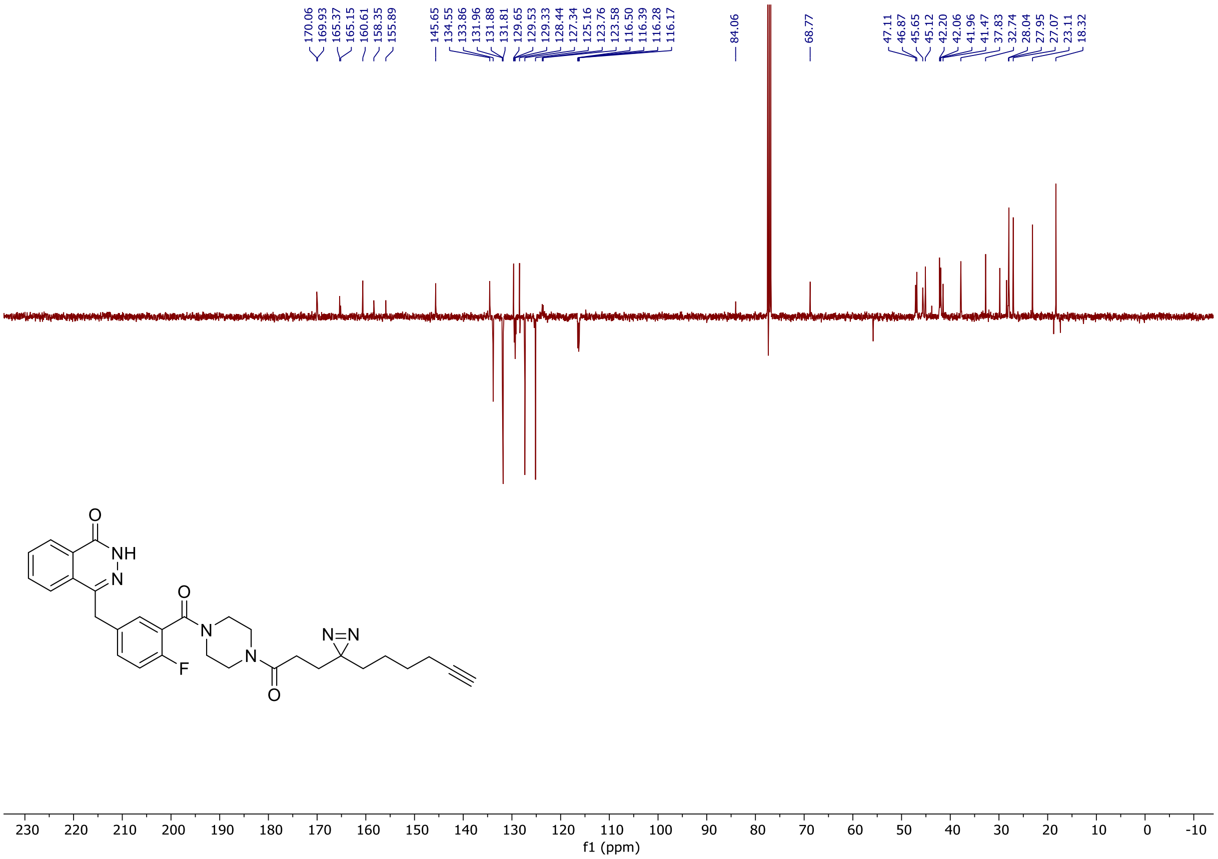

Figure S8. ^{13}C NMR Spectrum of Compound **7**

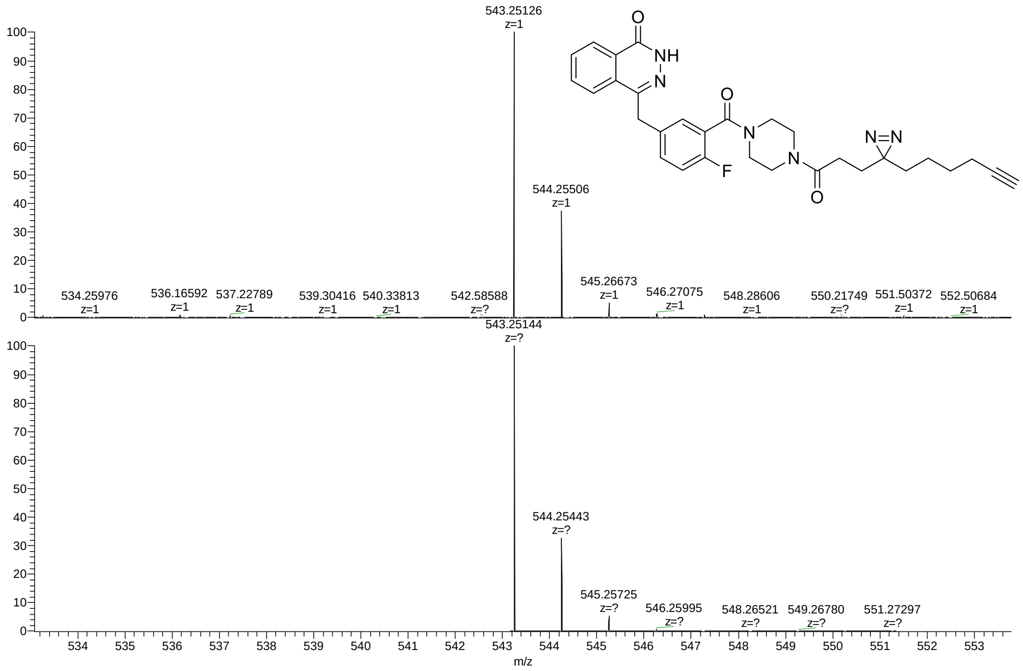

Figure S9. HRMS Data of Compound **7**

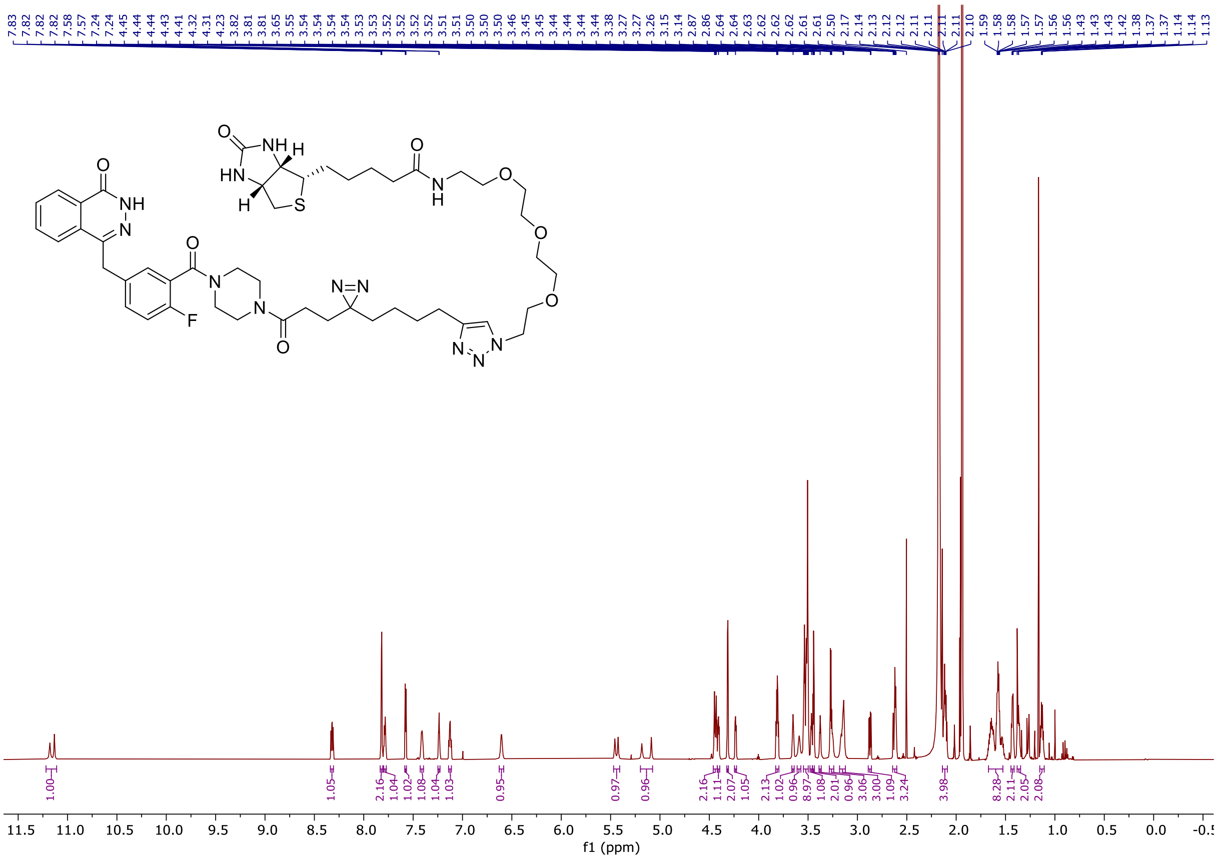

Figure S10. ^{1}H NMR Spectrum of Compound **4**

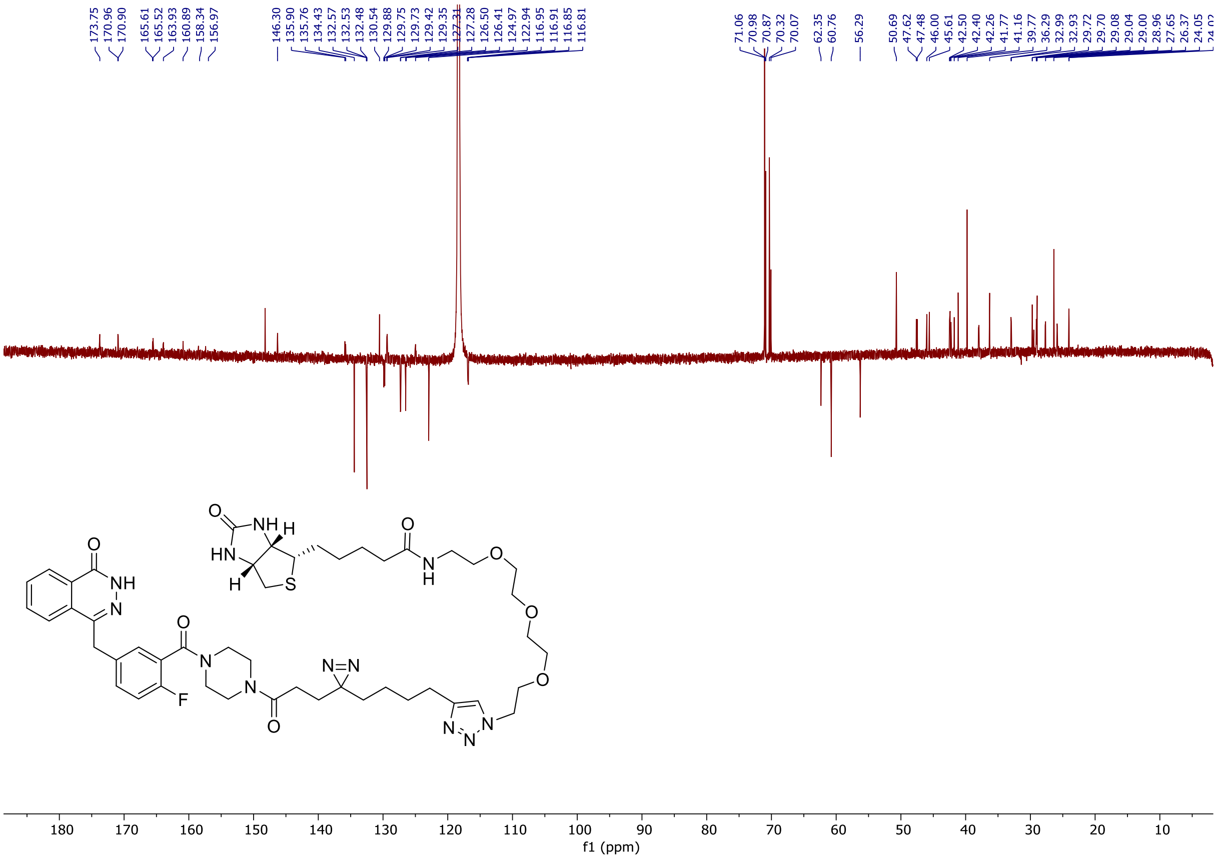

Figure S11. ^{13}C NMR Spectrum of Compound **4**

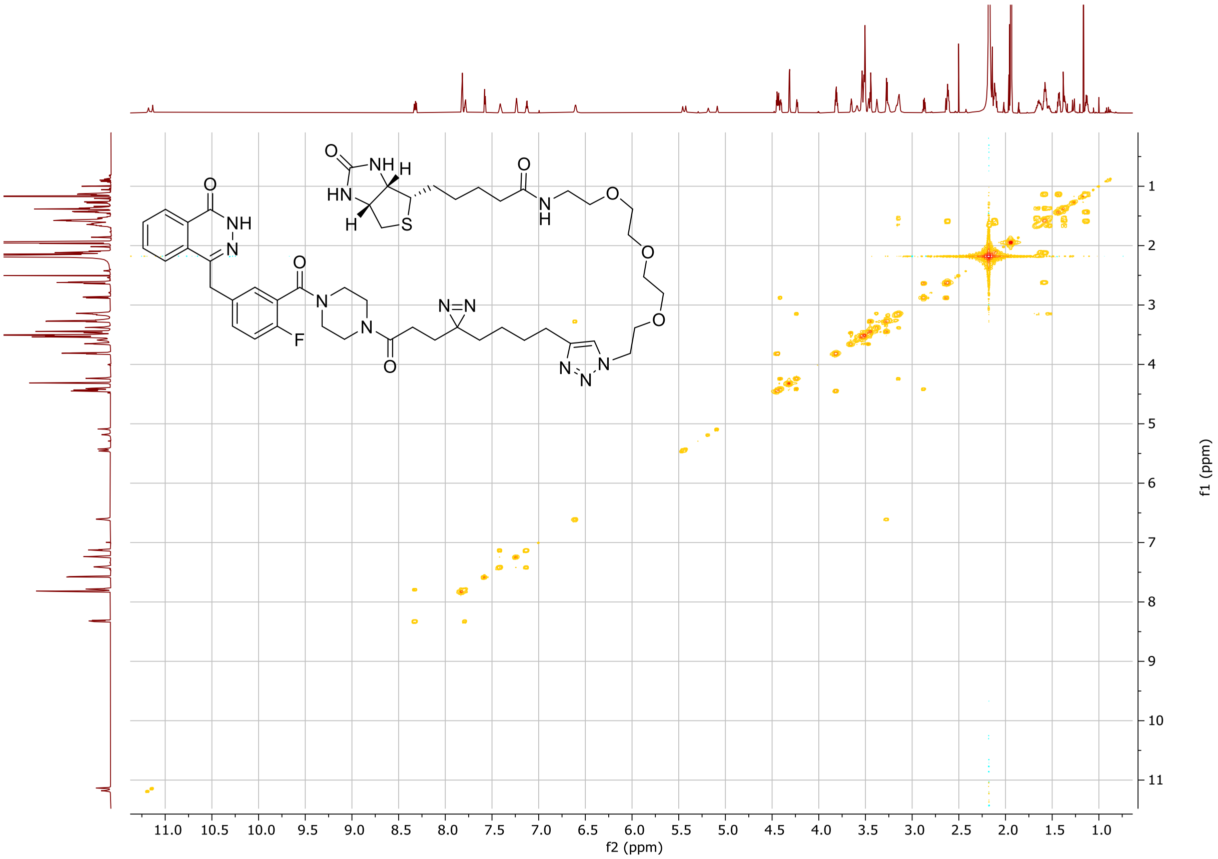

Figure S12. COSY Spectrum of Compound **4**

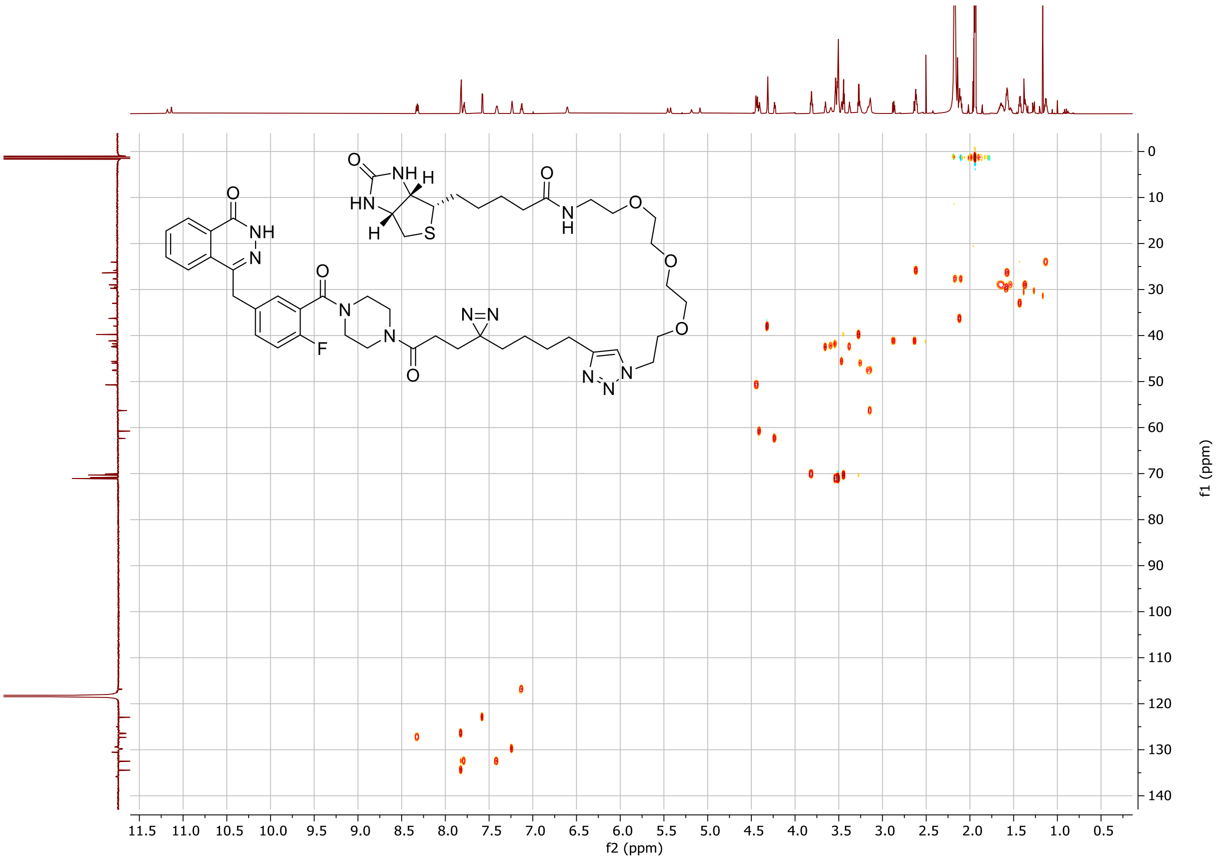

Figure S13. HSQC Spectrum of Compound **4**

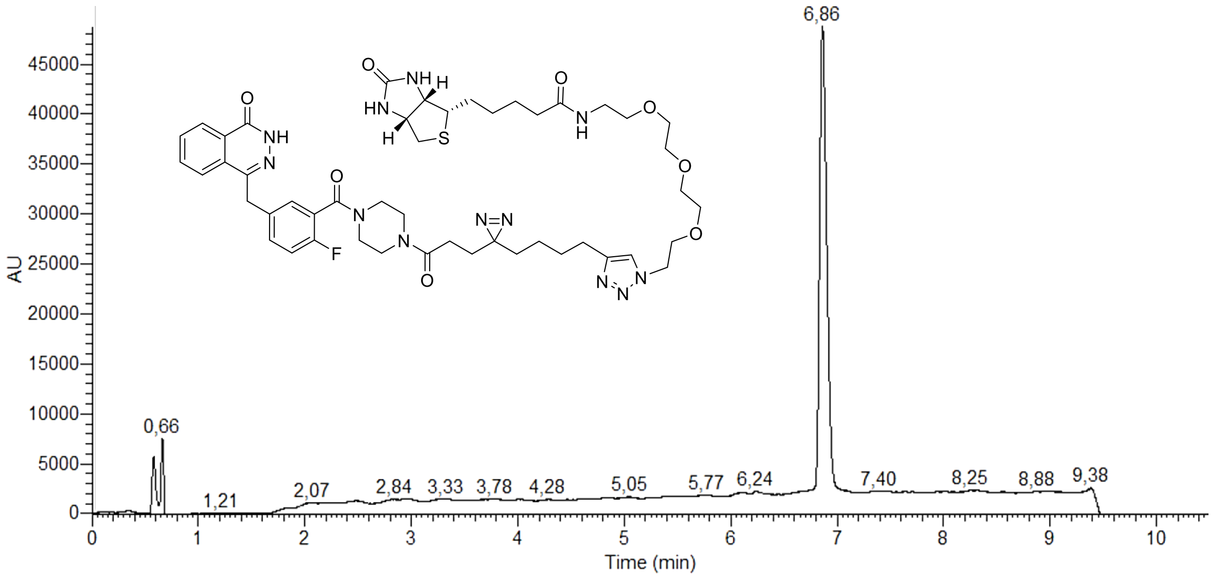

Figure S14. LCMS Analysis of Compound **4**

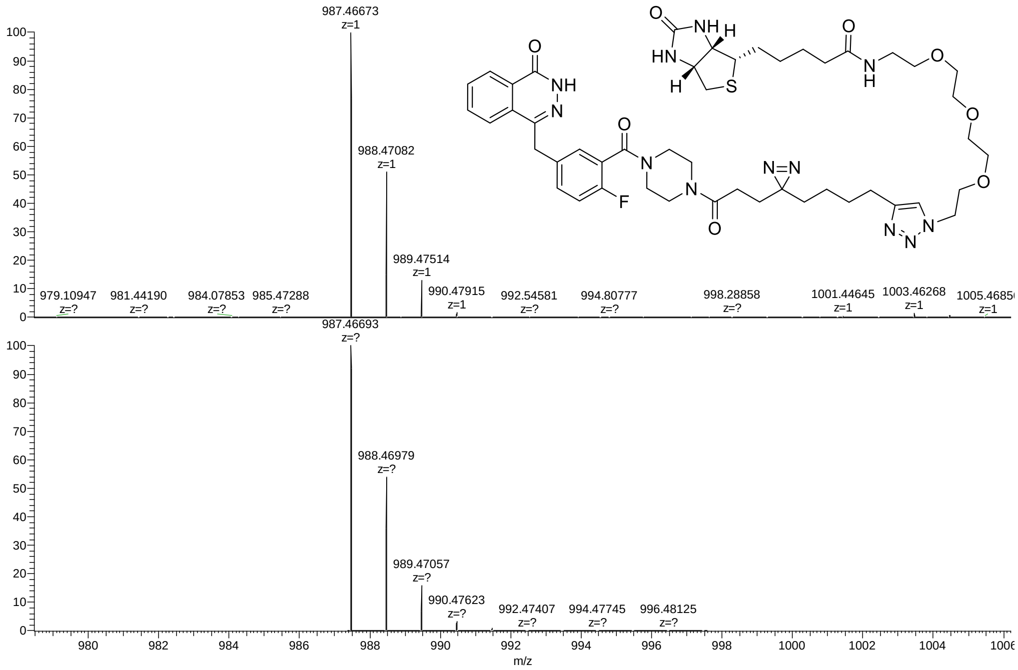

Figure S15. HRMS Data of Compound **4**

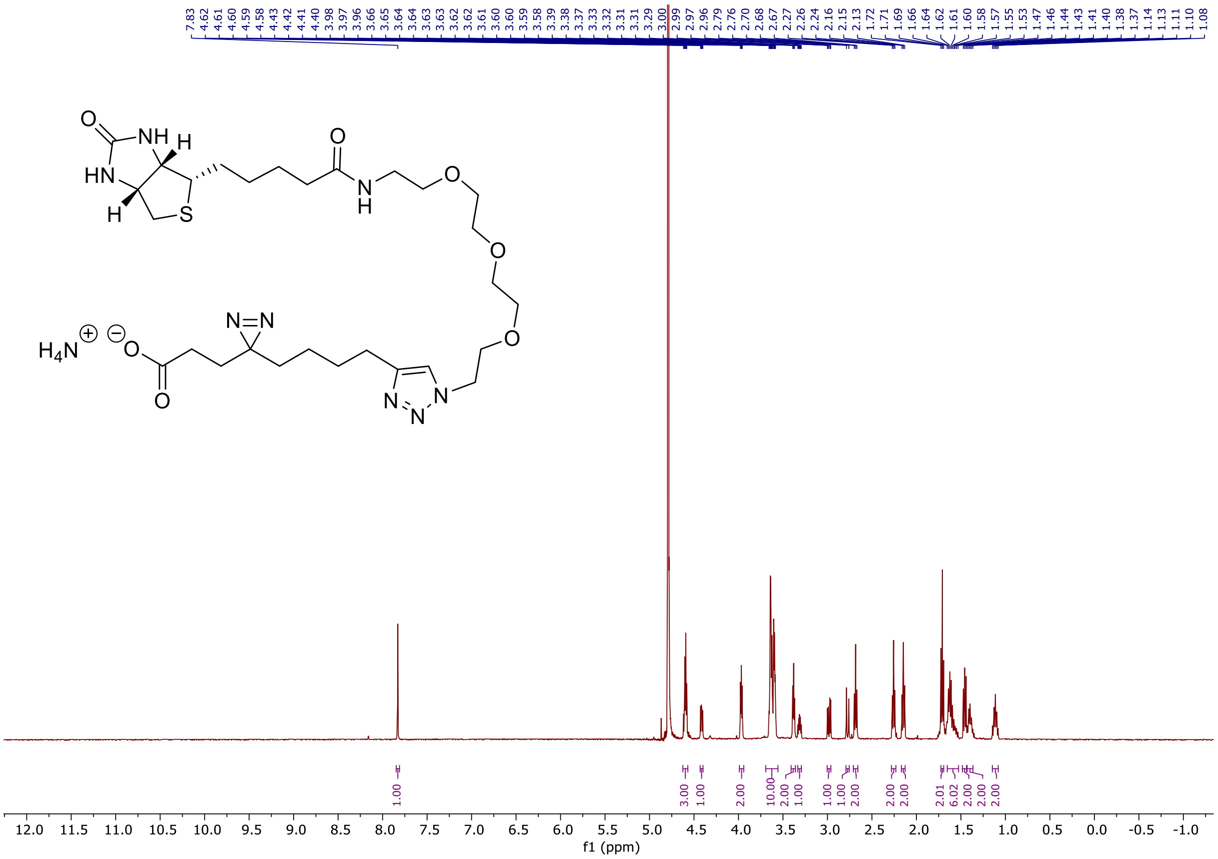

Figure S16. ^{1}H NMR Spectrum of Compound **9**

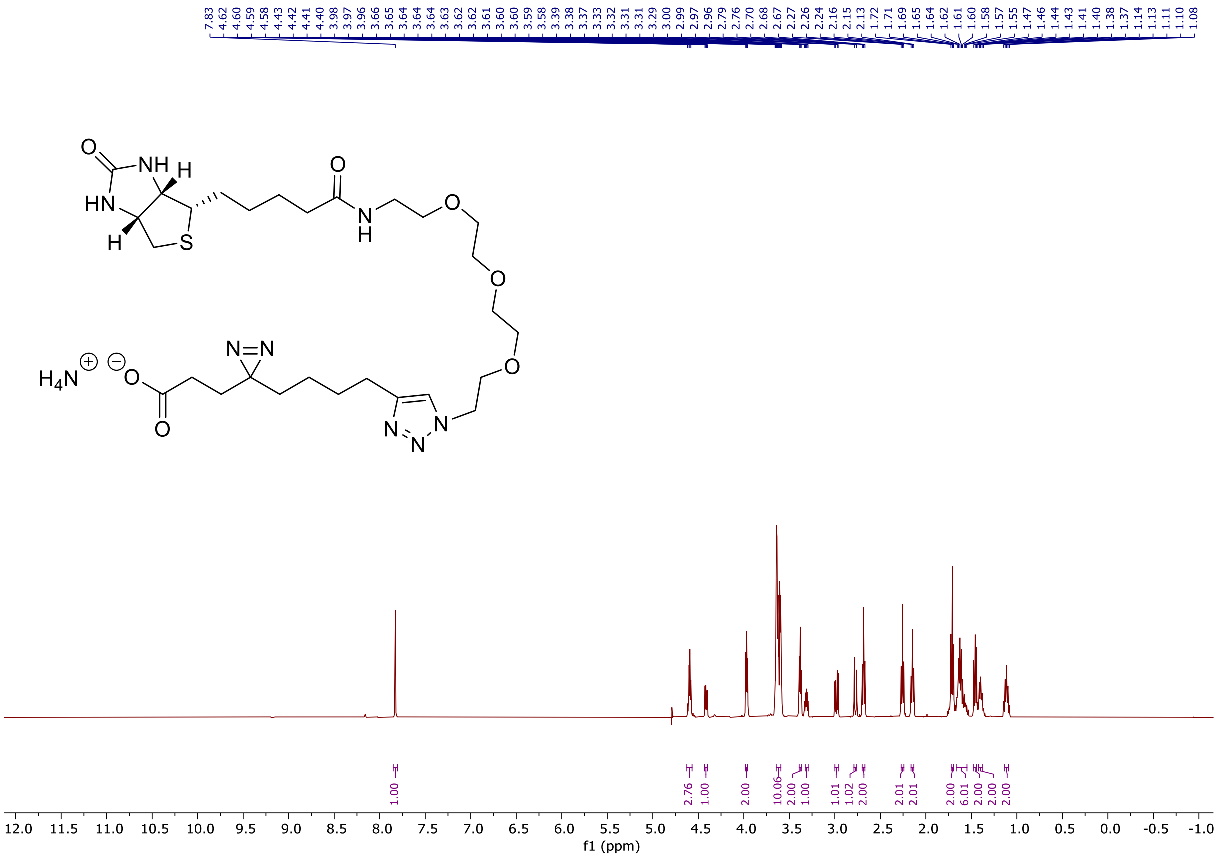

Figure S17. ^{1}H NMR Spectrum (presat) of Compound **9**

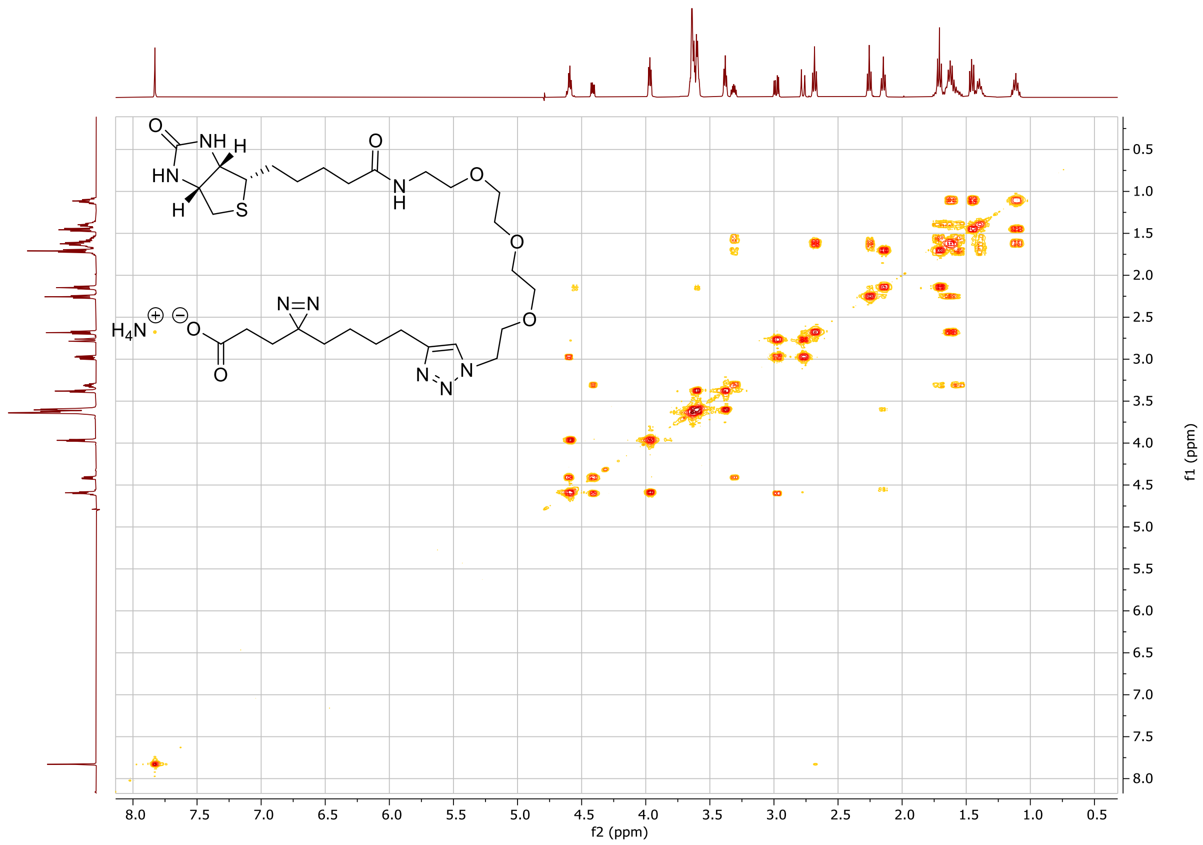

Figure S18. COSY Spectrum of Compound **9**

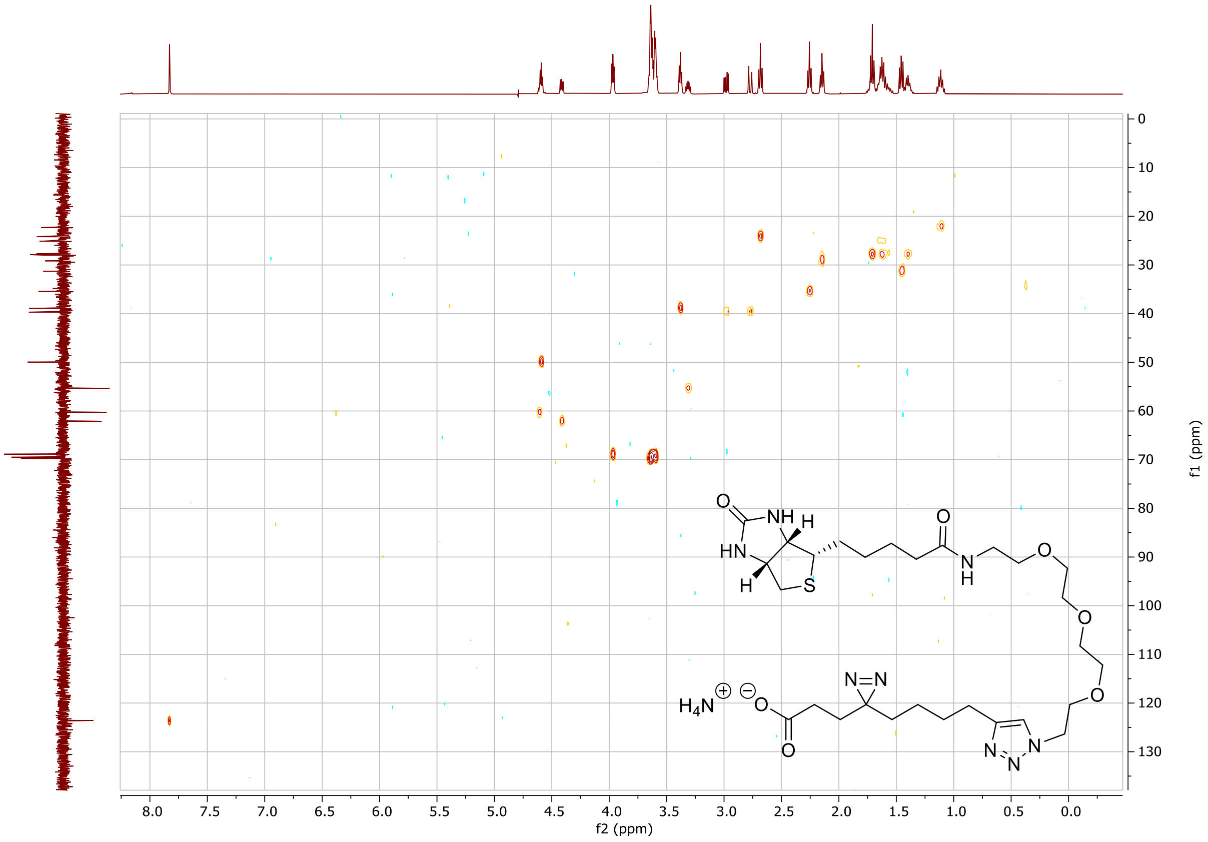

Figure S19. HSQC Spectrum of Compound **9**

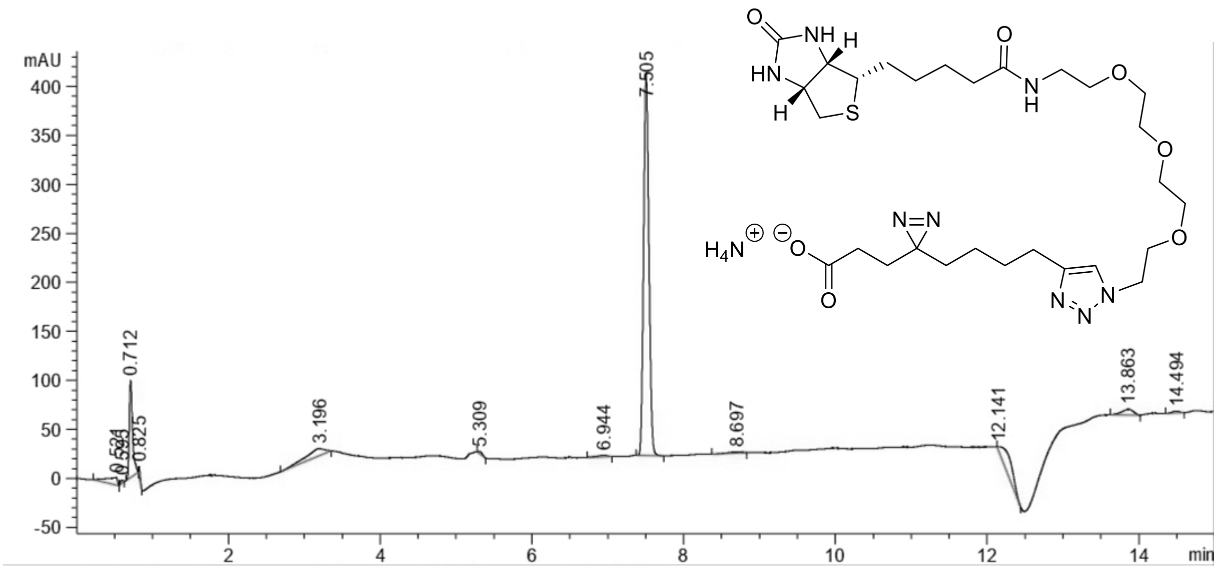

Figure S20. LCMS Analysis of Compound **9**

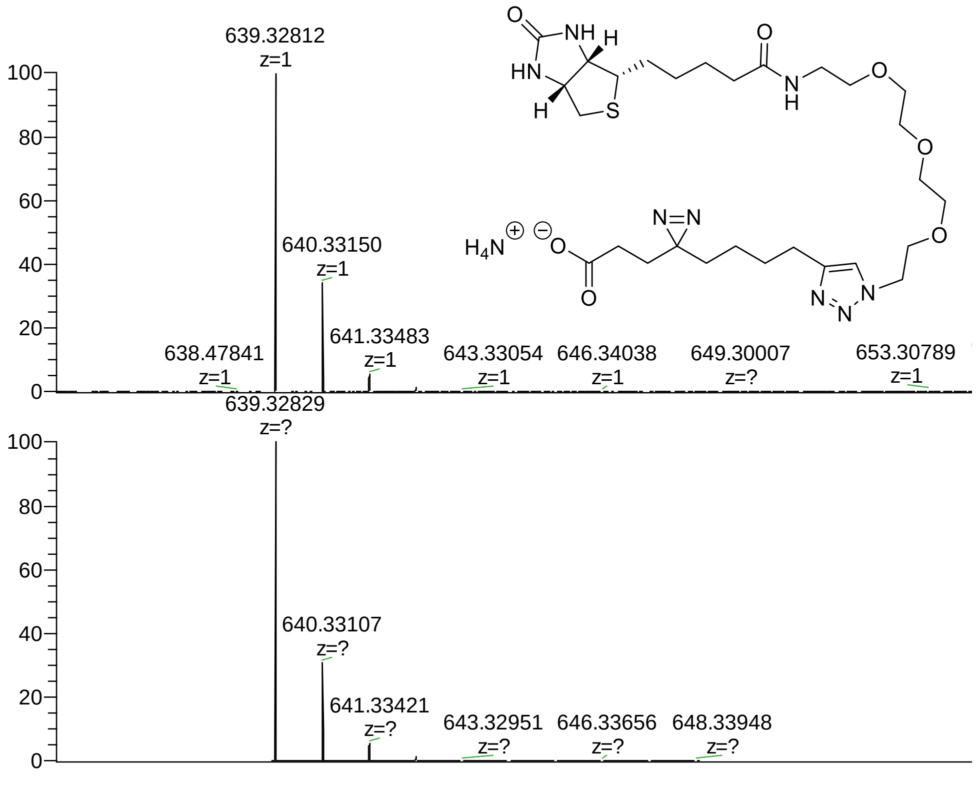

Figure S21. HRMS Data of Compound **9**
